# Environment-induced same-sex mating in the yeast *Candida albicans* through the Hsf1–Hsp90 pathway

**DOI:** 10.1371/journal.pbio.2006966

**Published:** 2019-03-13

**Authors:** Guobo Guan, Li Tao, Huizhen Yue, Weihong Liang, Jiao Gong, Jian Bing, Qiushi Zheng, Amanda O. Veri, Shuru Fan, Nicole Robbins, Leah E. Cowen, Guanghua Huang

**Affiliations:** 1 State Key Laboratory of Mycology, Institute of Microbiology, Chinese Academy of Sciences, Beijing, China; 2 University of Chinese Academy of Sciences, Beijing, China; 3 State Key Laboratory of Genetic Engineering, School of Life Sciences, Fudan University, Shanghai, China; 4 Department of Molecular Genetics, University of Toronto, Toronto, Ontario, Canada; UCSF, United States of America

## Abstract

While sexual reproduction is pervasive in eukaryotic cells, the strategies employed by fungal species to achieve and complete sexual cycles is highly diverse and complex. Many fungi, including *Saccharomyces cerevisiae* and *Schizosaccharomyces pombe*, are homothallic (able to mate with their own mitotic descendants) because of homothallic switching (HO) endonuclease-mediated mating-type switching. Under laboratory conditions, the human fungal pathogen *Candida albicans* can undergo both heterothallic and homothallic (opposite- and same-sex) mating. However, both mating modes require the presence of cells with two opposite mating types (*MTL***a/a** and α/α) in close proximity. Given the predominant clonal feature of this yeast in the human host, both opposite- and same-sex mating would be rare in nature. In this study, we report that glucose starvation and oxidative stress, common environmental stresses encountered by the pathogen, induce the development of mating projections and efficiently permit same-sex mating in *C*. *albicans* with an “**a**” mating type (*MTL***a/a**). This induction bypasses the requirement for the presence of cells with an opposite mating type and allows efficient sexual mating between cells derived from a single progenitor. Glucose starvation causes an increase in intracellular oxidative species, overwhelming the Heat Shock transcription Factor 1 (Hsf1)- and Heat shock protein (Hsp)90-mediated stress-response pathway. We further demonstrate that *Candida* TransActivating protein 4 (Cta4) and Cell Wall Transcription factor 1 (Cwt1), downstream effectors of the Hsf1–Hsp90 pathway, regulate same-sex mating in *C*. *albicans* through the transcriptional control of the master regulator of **a**-type mating, *MTL****a****2*, and the pheromone precursor-encoding gene Mating α factor precursor (*MF*α). Our results suggest that mating could occur much more frequently in nature than was originally appreciated and that same-sex mating could be an important mode of sexual reproduction in *C*. *albicans*.

## Author summary

*Candida albicans* is notorious as a human fungal pathogen that causes millions of incidents of thrush and systemic infection every year. Sexual reproduction plays a pivotal role in the biology and survival of pathogenic fungal pathogens. However, *C*. *albicans* is predominantly clonal, suggesting that mating and recombination between isolates would be rare in nature. Here, we report that environmental stresses induce the development of mating projections and efficient same-sex mating in *C*. *albicans*. This induction represents a novel mode of homothallism that is independent of the HO endonuclease-mediated mating-type switching observed in *Saccharomyces cerevisiae* and *Schizosaccharomyces pombe*. This represents a seminal example of how an environmentally relevant stress induces homothallic mating in fungi.

## Introduction

Sexual reproduction is a driving force for evolution and is prominent in eukaryotic organisms, with fungi adopting highly diverse strategies for sexual mating and reproduction [1, 2]. The human fungal pathogen *C*. *albicans* is a leading cause of death due to mycotic infection, with mortality rates approaching 40%, even with current treatments [3]. *C*. *albicans* has long been thought to be asexual until the discovery of a highly complex parasexual program. In *C*. *albicans*, heterothallic (opposite-sex) mating between diploid **a** and α cells occurs to generate tetraploid **a**/α intermediates [4, 5]. These tetraploid mating products undergo a parasexual process of concerted chromosome loss to generate diploid and aneuploid progeny rather than adopting a more traditional meiotic cycle [6, 7]. As an additional layer of complexity, an epigenetic switch from the white cell type to the opaque cell type is required for efficient mating to occur [8]. Opaque **a** and α cells secrete a sex-specific pheromone and induce the formation of mating projections in cells with an opposite Mating type locus (*MTL*) type, thus initiating cell fusion and mating [9]. Besides differences in mating competency, white and opaque cells also differ in a number of aspects, including metabolic profiles, filamentation ability, susceptibility to antifungals, interactions with host immune cells, and virulence in different infection models [10–12].

Given the predominately clonal nature of *C*. *albicans* [13, 14], the frequency with which heterothallic mating occurs in nature appears remarkably low. In the model yeast *S*. *cerevisiae*, most natural isolates are homothallic and able to undergo clonal mating because of the expression of homothallic switching (HO) endonuclease and mating-type switching [1]. However, *C*. *albicans* does not have a homolog of HO endonuclease and is unable to undergo mating-type switching and subsequent clonal mating [15]. Although same-sex mating (homothallism) has been reported in *C*. *albicans*, this unisexual mating has only been observed between two **a** cells, when α cells are present and secrete α-pheromone (ménage à trois mating), or in strains in which the BARrier 1 (Bar1) protease that degrades α-pheromone has been inactivated [16]. However, there is no evidence of natural ménage à trois mating, and no natural mutants of *BAR1* have been reported. Given the barriers to opposite- and same-sex mating, the biological relevance of sexual reproduction in *C*. *albicans* remains elusive.

Although *C*. *albicans* mating seems to be rare in nature, environmental stressors have been reported to promote loss of heterozygosity at the *MTL*, drive white-to-opaque switching, and result in concerted chromosome loss, suggesting that stress may be a trigger for sexual reproduction [17]. Nutrient starvation and oxidative stresses are two of the most common environmental stressors experienced by *C*. *albicans* in nature. Here, we establish that glucose starvation and oxidative stress efficiently induce the expression of sexual pheromone precursors, leading to the formation of mating projections and enabling homothallic mating in *C*. *albicans*. A core cellular stress-responsive pathway, mediated by the molecular chaperone Hsp90 and the heat-shock transcription factor Hsf1, is involved in this regulation through the downstream regulator Cwt1. Cwt1 functions through the direct control of the master regulator of **a**-type mating MTL**a**2, which regulates the expression of pheromone-encoding genes. Our study identifies a mechanism by which mating could occur much more frequently in nature than was originally appreciated, uncovers a core cellular stress-response pathway regulating this response, and sheds new light, to our knowledge, on the biology of *C*. *albicans*.

## Results

### Glucose starvation triggers the development of mating projections in *C*. *albicans*

**Fig 1:**
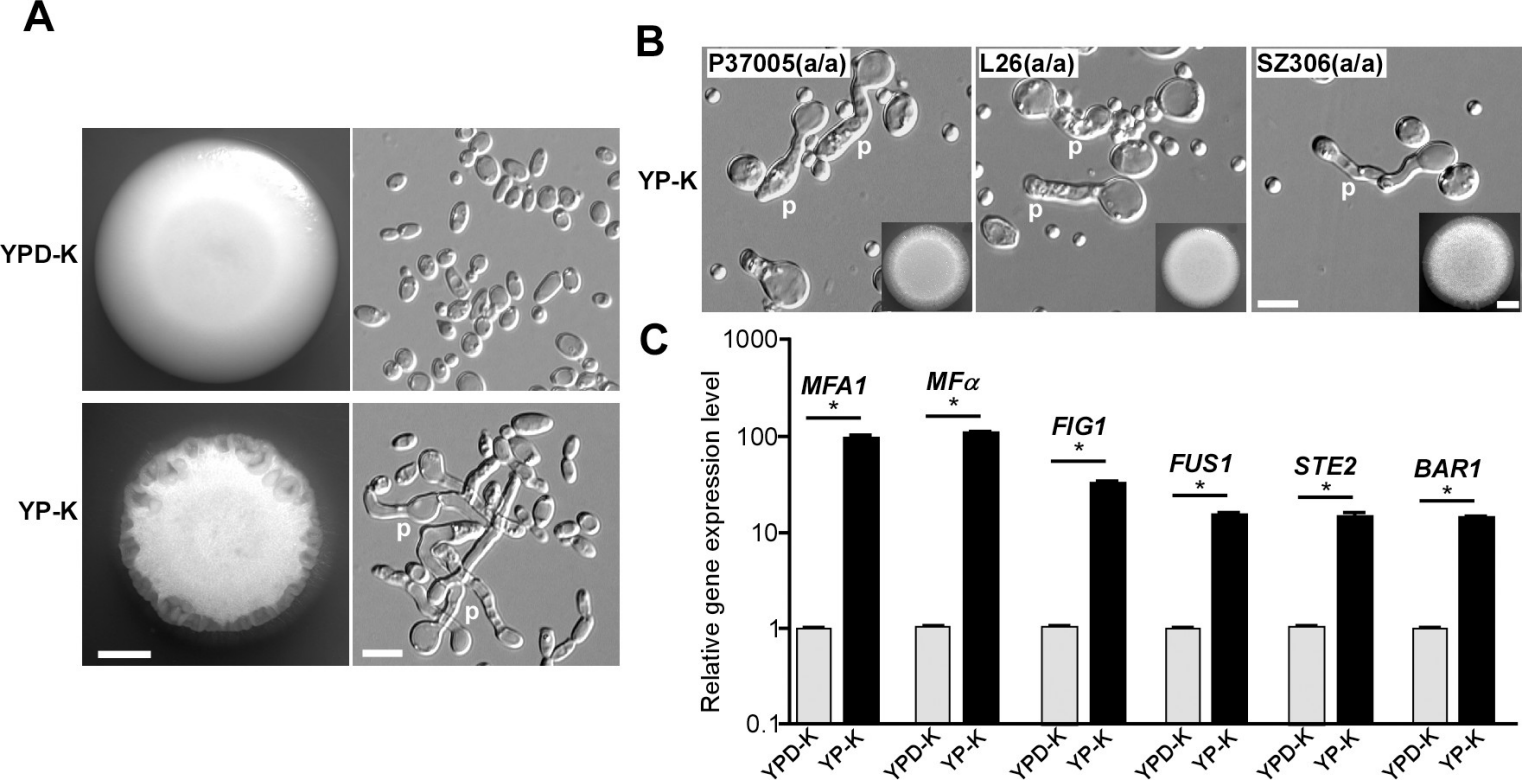
Glucose starvation promotes efficient polarized cell growth and induces the expression of mating-related genes in *MTL*a/a cells of *C*. *albicans*. (A) Morphologies of the laboratory strain GH1013 (*MTL***a**/**a**) grown on YPD-K and YP-K media. 1 × 10^5^ cells were spotted on YPD-K and YP-K media and cultured at 25°C for five days. Scale bar for colonies (left panels), 2 mm; scale bar for cells (right panels), 10 μm. (B) Morphologies of three clinical *C*. *albicans* strains (*MTL***a**/**a**) grown on YP-K medium at 30°C for four days. Scale bar for colonies (inset), 2 mm; scale bar for cells, 10 μm. (C) Relative expression levels of mating-related genes normalized to *ACT1*. Cells of GH1013 were used, and culture conditions were same as described in panel (A). Error bars represent standard errors of technical duplicates. *p < 0.05, two-tailed Student *t* test. Experiment was repeated in a biological replicate, and a representative image is shown. The numerical data are presented in **S3 Data**. *ACT1*, ACTin 1; Bar1, BARrier 1; *FIG1*, Factor-Induced Gene 1; *FUS1*, cell FUSion 1; *MFA1*, Mating type A1; *MF*α, Mating α factor precursor; *MTL*, Mating type locus; p, mating projection; *STE2*, STErile 2; YPD-K, yeast extract-peptone-glucose-K_2_HPO_4_; YP-K, yeast extract-peptone-K_2_HPO_4_.

Despite the fact that cellular stressors promote loss of heterozygosity at the *MTL* and induce the white-to-opaque switch [17, 18], there still remains no evidence to directly support the hypothesis that environmental perturbations regulate sexual mating in *C*. *albicans*. In the human host, *C*. *albicans* resides on mucosal surfaces, niches that are often glucose limited [19]. Therefore, we cultured *C*. *albicans* cells in the absence of glucose but in the presence of 0.25% K_2_HPO_4_, which acts as a pH-buffering reagent. We named this modified medium as YP-K (1% yeast extract, 2% peptone, 0.25% K_2_HPO_4_, w/v) and the control medium as YPD-K (1% yeast extract, 2% peptone, 2% glucose, 0.25% K_2_HPO_4_, w/v). Opaque cells were used for all experiments in this study because they are the mating-competent form in *C*. *albicans* [8]. Upon spotting an *MTL***a/a** *C*. *albicans* strain (GH1013) [20], we noted a wrinkled colony morphology on YP-K agar after five days of growth (**Fig 1A**). Surprisingly, a portion of cells underwent polarized cell growth (**Fig 1A**) that was elongated and irregular in shape, closely resembling mating projections as opposed to the typical hyphal morphology [21]. When the same strain was cultured on the glucose-containing YPD-K medium, the colonies remained smooth, and cells were exclusively in the yeast form (**Fig 1A**). This was a dose-dependent effect because incremental increases in glucose levels resulted in incremental decreases in the number of polarized cells observed (**S1 Fig**). We also observed the development of polarized cells on YP-K medium for three independent clinical isolates of *C*. *albicans* with an *MTL***a/a** genotype (P37005, L26, and SZ306) (**Fig 1B**), suggesting that this inducing effect of glucose starvation is a general feature in *MTL***a/a** strains. The induction of mating projections was not observed in any *MTL***a**/α and *MTL*α/α strains under the same culture conditions.

To verify that the elongated cells were true mating projections but not pseudohyphae or true hyphae, we examined the relative expression of mating-related genes using quantitative reverse transcription PCR (qRT-PCR) (**Fig 1C**). Compared to the YPD-K cultures, the relative expression levels of Mating type A1 (*MFA1*) (a precursor of **a**-pheromone), *MFα* (a precursor of α-pheromone), STErile 2 (*STE2*) (the α-pheromone receptor), and Factor-Induced Gene 1 (*FIG1*) and cell FUSion 1 (*FUS1*) (two pheromone-response genes), as well as *BAR1* (an endopeptidase that degrades α-pheromone), were all significantly increased in cells grown on YP-K. We also constructed four reporter strains in which *MFA1*, *MFα*, *FIG1*, and *FUS1* were fused with a green fluorescent protein (GFP) fluorescent marker to further confirm their increased expression on YP-K medium (**S2 Fig**). Thus, glucose depletion leads to the induction of mating-related genes and polarized cell growth, indicative of the development of mating projections in *C*. *albicans*.

### Glucose starvation promotes same-sex mating in *C*. *albicans*

**Fig 2:**
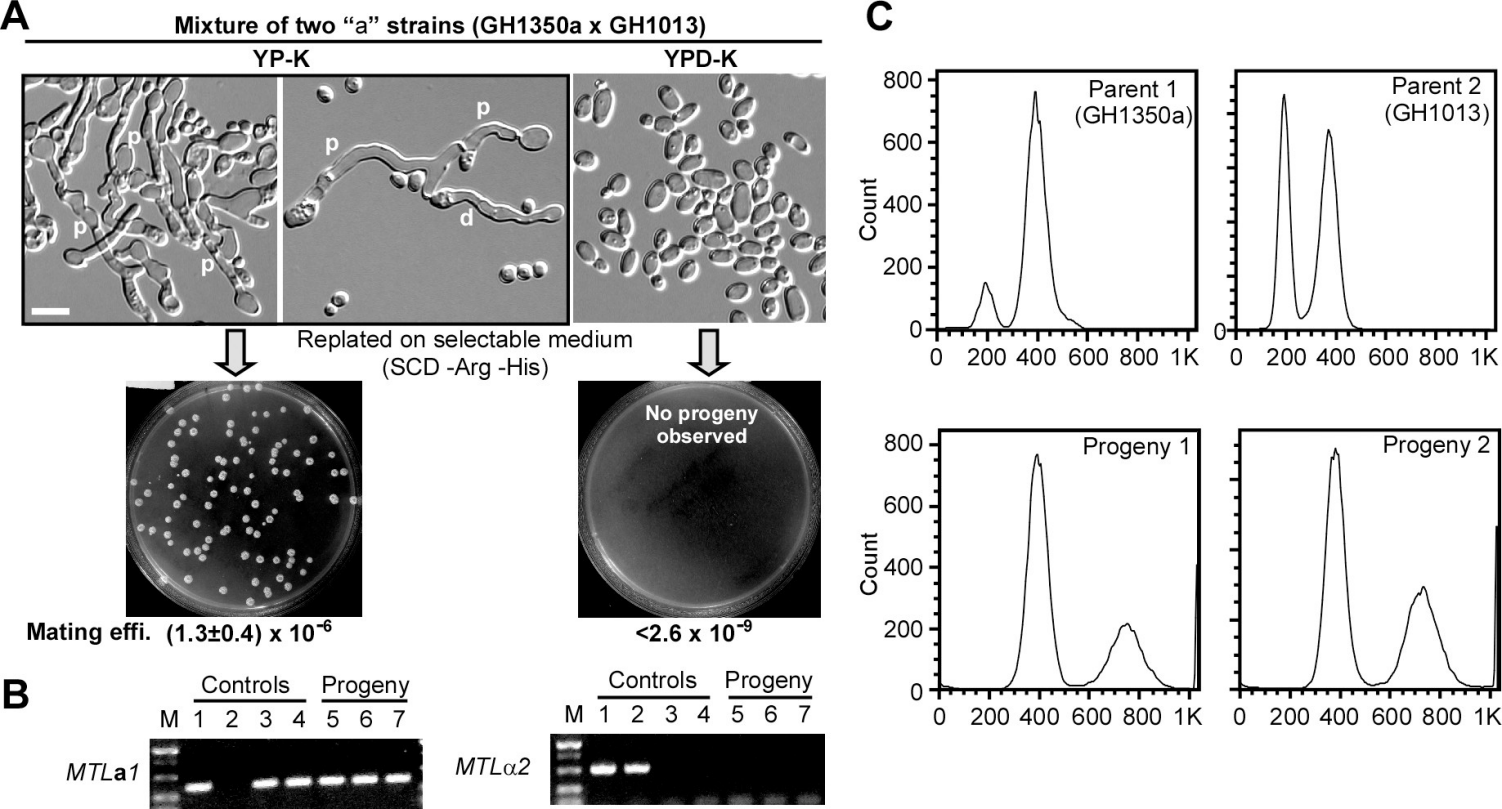
Glucose starvation induces same-sex mating in *C*. *albicans*. (A) Same-sex mating between two “**a**” strains (GH1013 and GH1350a). 1 × 10^7^ cells of each strain were mixed and cultured on YPD-K and YP-K media at 25°C. After three days of growth, the mating mixture was replated onto SCD-Arg, SCD-His, and SCD-Arg-His dropout plates to assess mating efficiency. The middle panel (up) indicates that two “**a**” cells underwent cell fusion and a daughter cell grew out from the conjunction tube. Scale bar, 10 μm. (B) PCR verification of the *MTL* types. Strains used: lane 1, SN152 (**a**/α); 2, GH1350 (α/α); 3, GH1350a (**a**/**a**); 4, GH1013 (**a**/**a**); 5–7, progeny strains of the GH1350a × GH1013a cross. (C) FACS analysis of the DNA content of parental and progeny strains. Parental diploids have the standard G1 and G2 cell cycle peaks representing 2C and 4C DNA levels. Mating progeny contain DNA content corresponding to 4C and 8C peaks, confirming their tetraploid nature. Arg, arginine; d, daughter cell; FACS, fluorescence-activated cell sorting; His, histidine; M, DNA ladder; *MTL*, Mating type locus; p, mating projection; SCD, synthetic complete medium; YPD-K, yeast extract-peptone-glucose-K_2_HPO_4_; YP-K, yeast extract-peptone-K_2_HPO_4_.

The induction of both **a**-pheromone and α-pheromone precursors in *MTL***a/a** cells under glucose-starvation conditions suggests those cells might have lost their original sexual identity, exhibiting features of both *MTL***a/a** and *MTL*α/α cells. This, in theory, could bypass the requirement of an *MTL*α/α cell in close proximity to induce homothallic mating between two *MTL***a/a** partners. To further explore whether the induction of mating-related genes and polarized cell growth could lead to true homothallic mating, we performed quantitative same-sex mating assays. When two *MTL***a/a** strains with different auxotrophic markers (GH1350a and GH1013) were cultured together on YP-K medium, mating projections were observed (**Fig 2A**), and tetraploid progeny that remained the **a** mating type were generated (**Fig 2B and 2C)**, confirming homothallic mating had occurred. Further, cell fusion between two *MTL***a/a** cells and the generation of daughter cells were also observed on YP-K medium (**Fig 2A**). In contrast, cells remained in yeast form and no mating progeny were isolated when the two *MTL***a/a** strains were grown on YPD-K (**Fig 2**). These results provide the premier example of an environmentally relevant stress, glucose starvation, capable of inducing same-sex mating in *C*. *albicans*.

### Multiple environmental stressors mediate the development of mating projections in *C*. *albicans*

**Fig 3:**
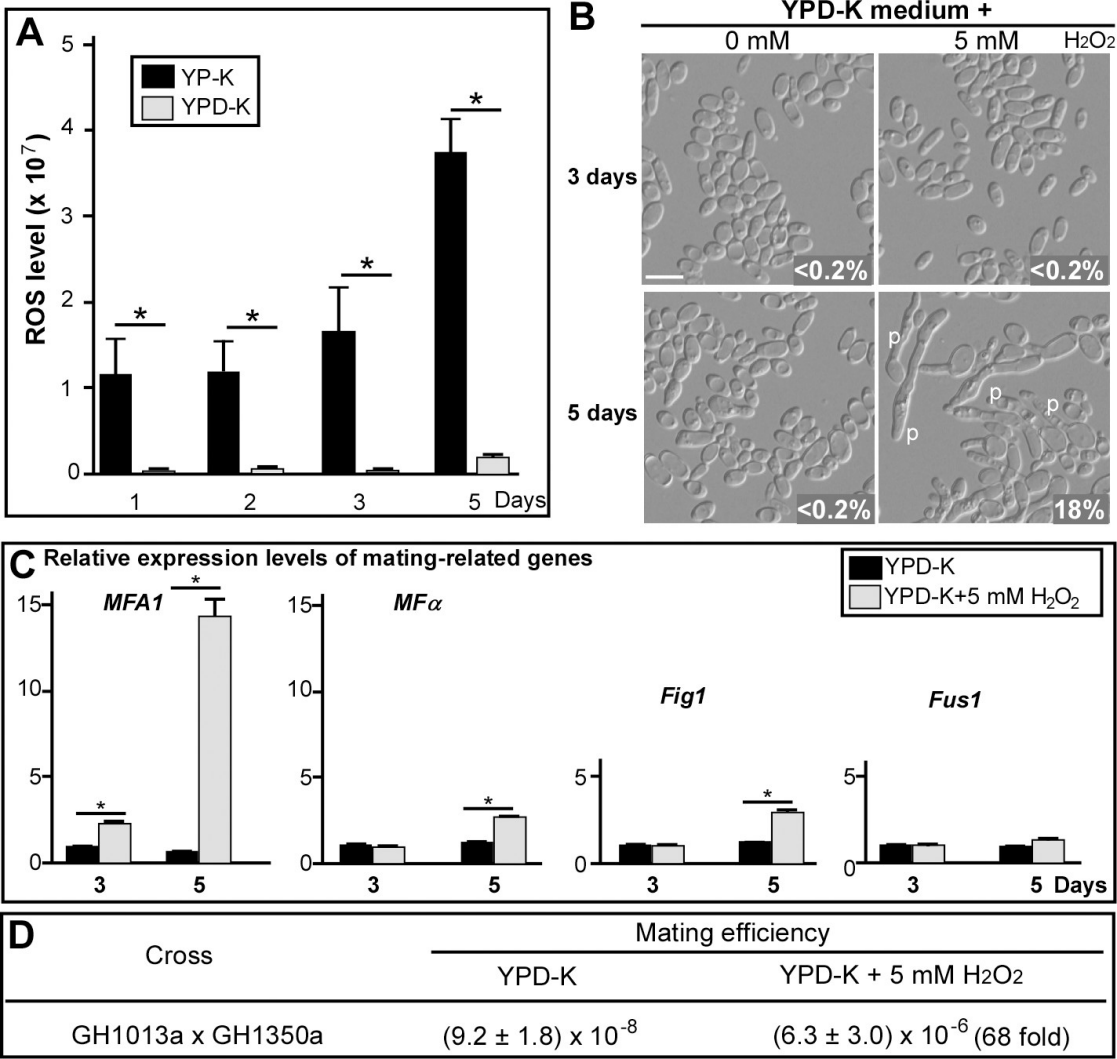
Oxidative stress promotes the development of mating projections in *C*. *albicans*. (A) Relative ROS levels. 1 × 10^5^ cells of strain GH1013 were spotted on YPD-K and YP-K media and cultured at 25°C for one, two, three, or five days. For each day, 1 × 10^6^ cells were used for ROS level determination. Error bars represent standard deviation of three biological replicates. * indicates significant difference (*p* < 0.05, two-tailed Student *t* test). (B) H_2_O_2_ induces the development of mating projections in the presence of glucose. 200 μL of H_2_O or 5 mM H_2_O_2_ solution was spread on YPD-K medium plates (90 mm). 1 × 10^6^ cells of strain GH1350a were spotted on the medium and cultured at 25°C for three or five days. Percentages of projected cells are indicated in the corresponding images. (C) Relative expression levels of mating-related genes in response to H_2_O_2_ treatments on YPD-K medium. Error bars, standard errors of technical duplicates. *p < 0.05, two-tailed Student *t* test. Experiment was performed in biological replicate, and a representative image is shown. (D) Efficiency of same-sex mating on YPD-K medium with or without H_2_O_2_ treatment. The mating mixtures were grown on different media at 25°C for seven days. To make YPD-K + H_2_O_2_ plates, 200 μL H_2_O_2_ (5 mM) was spread on the medium surface (of a 90-mm plate). The numerical data are presented in **S3 Data**. *FIG1*, Factor-Induced Gene 1; *FUS1*, cell FUSion 1; *MFA1*, Mating type A1; *MF*α, Mating α factor precursor; p, mating projection; ROS, reactive oxidative species; YPD-K, yeast extract-peptone-glucose-K_2_HPO_4_; YP-K, yeast extract-peptone-K_2_HPO_4_.

Glucose deprivation leads to a shift in *C*. *albicans* metabolism, resulting in the activation of genes involved in the tricarboxylic acid (TCA) cycle and fatty acid β-oxidation [19]. These metabolic changes stimulate the production of reactive oxidative species (ROSs) that cause protein damage, thereby activating the unfolded protein response [22]. The intracellular levels of ROSs in cells of *C*. *albicans* grown on YP-K medium were significantly higher than those on YPD-K medium and increased with the extension of culture time (**Fig 3A**). To assess whether oxidative stress could stimulate homothallic mating, *C*. *albicans* cells were treated with hydrogen peroxide (H_2_O_2_), a strong oxidative-stress–inducing agent. Compared to the untreated control, H_2_O_2_-treated cells underwent obvious polarized cell development on the glucose-containing medium YPD-K (**Fig 3B**). Mating-related genes (*MFA1*, *MFα*, and *FIG1*) were significantly induced in H_2_O_2_-treated cells (**Fig 3C**), and mating efficiency upon H_2_O_2_ treatment was comparable to that observed under glucose-deprivation conditions (**Fig 3D**).

To further test other environmentally relevant stress conditions, we performed same-sex mating assays using three other nutrient-poor media types: 3% agar with no additional nutrients, agar containing 3% mouse feces, and agar containing *C*. *albicans* debris. *C*. *albicans* underwent same-sex mating when cultured on these media types, with mating on mouse-feces–containing medium being most efficient (**S3 Fig**). Notably, although mating projections morphologically resemble filaments in *C*. *albicans*, same-sex mating did not occur when cells were cultured on sorbitol medium that induces opaque cell filamentation (**S3E Fig**). Therefore, multiple nutrient-deprivation conditions are capable of inducing homothallic mating in *C*. *albicans*.

### Glucose starvation induces the expression of mating-related genes, stress-responsive genes, and *HSP90* genetic interactors

**Fig 4:**
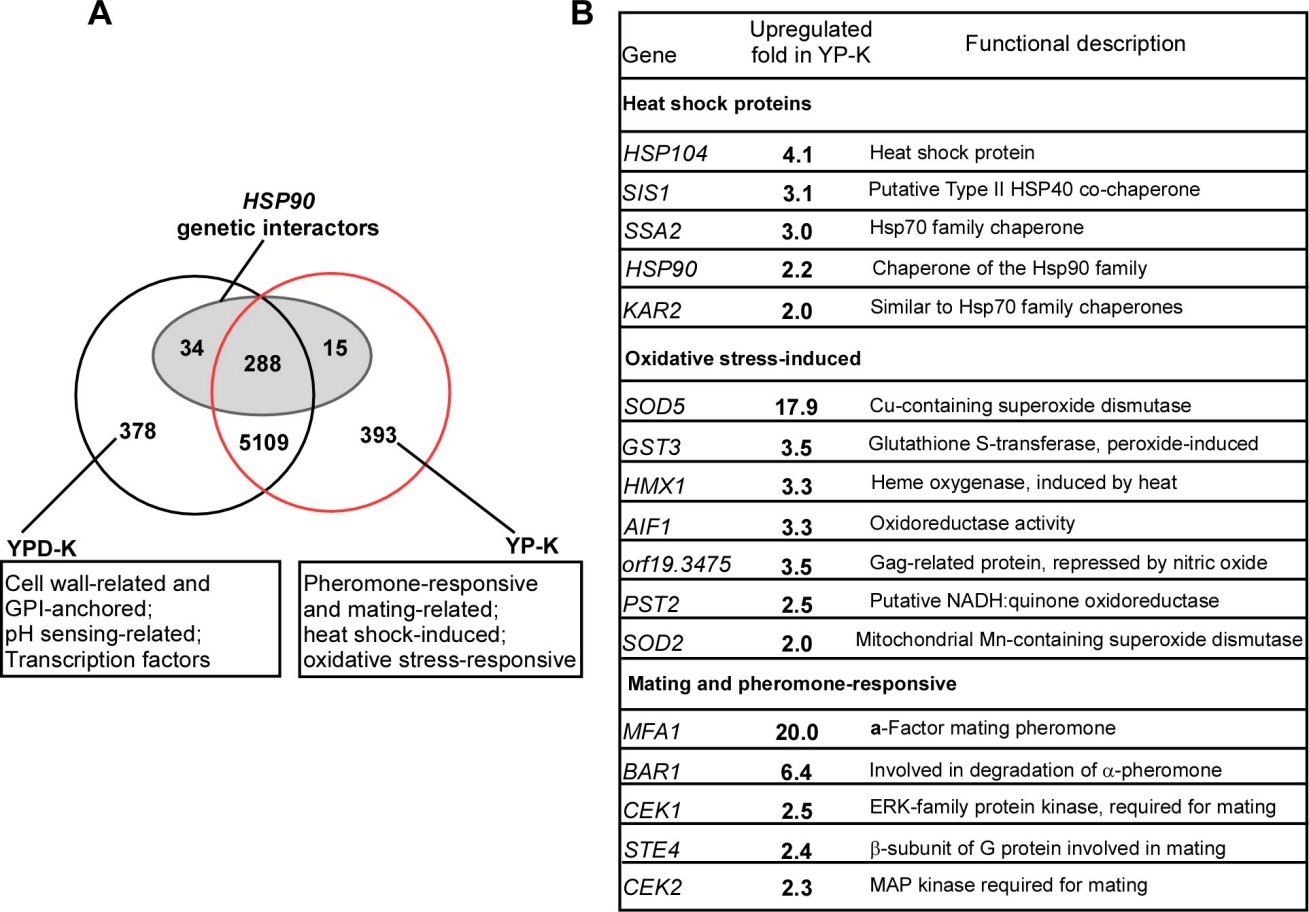
Global gene-expression–profile analysis of *C*. *albicans* in the presence and absence of glucose. *C*. *albicans* cells were spotted and grown on YPD-K or YP-K media at 25°C for 60 hours. Total RNA was extracted and used for RNA-Seq assays. To be considered differentially expressed, a gene must satisfy three criteria: (1) an FPKM value higher than or equal to 20 at least in one sample, (2) a fold change value higher than or equal to 1.5, and (3) an adjusted *p*-value (FDR) lower than 0.05. (A) Venn diagram depicting relationships between differentially expressed genes on YPD-K (412) and YP-K (408) media and *HSP90* genetic interactors (indicated in the ellipse). (B) Selected heat-shock-protein–encoding, oxidative-stress–induced, and mating-related genes up-regulated in YP-K medium. *AIF1*, Apoptosis-Inducing Factor 1; *BAR1*, BARrier 1; *CEK1*, *Candida* ERK-family protein kinase; ERK, ERK-family protein kinase; FDR, false discovery rate; FPKM, fragments per kb per million reads; GPI, glycosylphosphatidylinisotol; *GST3*, Glutathione S-transferase; *HMX1*, HeMe oXygenase; Hsp90, Heat shock protein 90; *KAR2*, KARyogamy; MAP, mitogen-activated protein; *MFA1*, Mating type A1; NADH, Nicotinamide adenine dinucleotide; *orf19*.*3475*, *Candida* gene *orf19*.*3475*; *PST2*, Protoplasts-SecreTed 1; RNA-Seq, RNA sequencing; *SIS1*, Slt4 Suppressor; *SOD5*, SuperOxide Dismutase 5; *SSA2*, Stress-Seventy subfamily A; *STE4*, STErile 4; YPD-K, yeast extract-peptone-glucose-K_2_HPO_4_; YP-K, yeast extract-peptone-K_2_HPO_4_.

To explore the mechanism of glucose-induced same-sex mating in *C*. *albicans*, we performed global gene-expression–profile analysis. Total RNA was extracted from opaque cells grown on YPD-K or YP-K media at 25°C for 60 hours, and the samples collected were used for RNA sequencing (RNA-Seq) assays. We incubated cells for only 60 hours because mating projections were not induced at this time point, enabling us to minimize indirect effects on gene expression. As demonstrated in **Fig 4** and **S1 Data**, we found 412 genes up-regulated in YPD-K medium and 408 genes up-regulated in YP-K medium (with a change greater than 1.5-fold). As expected, a number of mating-related genes—including *MFA1*, *BAR1*, *Candida* ERK-family protein kinase (*CEK*)*1*, and *CEK2*—were up-regulated in YP-K medium. Heat-shock-protein–encoding genes and oxidative-stress–responsive genes were also up-regulated in YP-K medium, whereas cell-wall–related and glycosylphosphatidylinisotol (GPI)-anchored-protein–encoding genes were enriched in YPD-K medium. Of the differentially expressed genes, 49 have been reported as *HSP90* genetic interactors (**Fig 4A** and **S2 Data**) [23, 24]. Among the differentially expressed *HSP90* genetic interactors, 34 genes were down-regulated and only 15 genes were up-regulated in YP-K medium. These genes were enriched in gene functions associated with stress response, cell wall, transcriptional regulation, and signaling transduction.

### Down-regulation of Hsf1–Hsp90 signaling promotes mating-projection formation and same-sex mating

Several observations led us to hypothesize that Hsp90 could play an important role in the regulation of glucose-starvation–induced mating-projection formation and same-sex mating. First, glucose starvation increased the intracellular level of ROSs (**Fig 3A**) that would contribute to increased protein damage, thus likely overwhelming the functional capacity of Hsp90. Second, the treatment of oxidative reagents (e.g., H_2_O_2_) that led to the increase of intracellular ROSs induced mating-projection formation and same-sex mating in *C*. *albicans* (**Fig 3B**, **3C** and **3D**). Third, a number of heat-shock-protein–encoding genes, including *HSP90*, were up-regulated in the absence of glucose, further suggesting that Hsp90 functional capacity was overwhelmed (**Fig 4B** and **S1 Data**).

**Fig 5:**
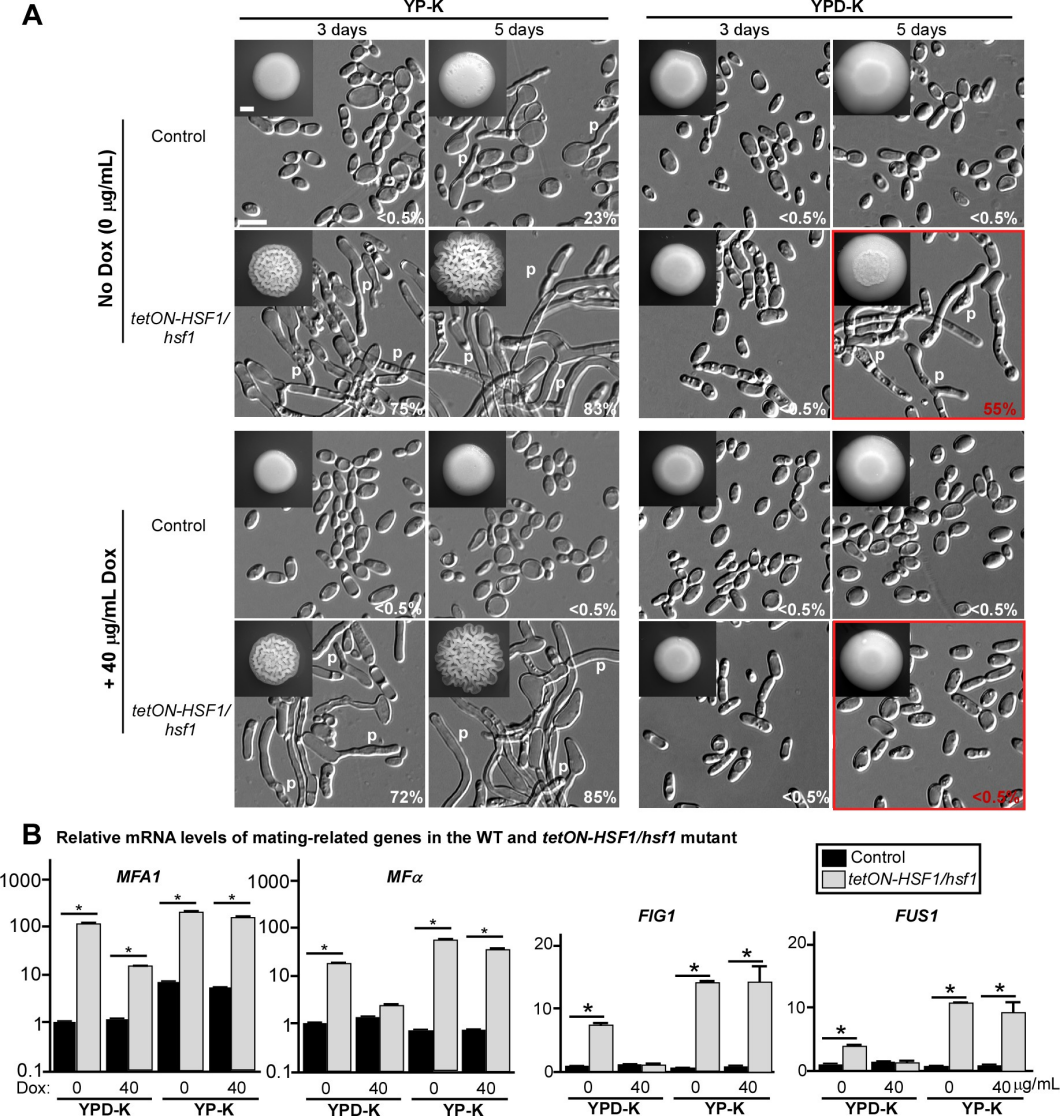
Role of Hsf1 in the induction of mating projections in *C*. *albicans*. (A) Morphologies of the control and *tetON-HSF1/hsf1* mutant. 1 × 10^5^ cells were spotted on YPD-K and YP-K media without or with 40 μg/mL Dox and cultured at 25°C for three or five days. Percentages of projected cells are indicated in the corresponding images. Scale bar for colonies, 2 mm (inset); scale bar for cells, 10 μm. Control, GH1013cartTA. (B) Relative expression levels of mating-related genes. 1 × 10^5^ cells of each strain were cultured on YPD-K medium (for six days) or on YP-K medium (for three days) without or with 40 μg/mL Dox at 25°C. Error bars represent standard errors of technical duplicates. *p < 0.05, two-tailed Student *t* test. Experiment was repeated in a biological replicate, and a representative image is shown. The numerical data are presented in **S3 Data**. Dox, doxycycline; *FIG1*, Factor-Induced Gene 1; *FUS1*, cell FUSion 1; Hsf1, Heat Shock transcription Factor 1; *MFA1*, Mating type A1; *MF*α, Mating α factor precursor; p, mating projection; *tetON*, tetracycline-induced; *tetON-HSF1/hsf1*, *tetON*-promoter–controlled conditional expression strain of *HSF1*; WT, wild type; YPD-K, yeast extract-peptone-glucose-K_2_HPO_4_; YP-K, yeast extract-peptone-K_2_HPO_4_.

The conserved heat-shock transcription factor Hsf1 plays an important role in regulating the expression of heat-shock proteins, including the molecular chaperone Hsp90 in *C*. *albicans* and other eukaryotes [25, 26]. Hsf1 has been implicated in regulating transcriptional changes in response to oxidative stresses and glucose starvation in *S*. *cerevisiae* [27, 28]. Therefore, we first tested the role of Hsf1 in the development of mating projection and homothallic mating in *C*. *albicans*. Since Hsf1 is essential for cell viability, we generated a tetracycline-induced (*tetON*)-promoter–controlled conditional expression mutant, *tetON-HSF1/hsf1*, in order to assess how changes in Hsf1 levels influence same-sex mating. In the absence or presence of 40 μg/mL doxycycline, the *tetON-HSF1/hsf1* mutant formed wrinkled colonies and underwent robust polarized growth on YP-K medium (**Fig 5A**). Of note, doxycycline (40 μg/mL) exhibited an inhibitory effect on the induction of mating projection formation in the wild-type (WT) control (**Fig 5A**). With increased exposure to glucose on YPD-K medium, mating projections were only observed in the absence of doxycycline after five days but not upon the addition of 40 μg/mL doxycycline nor in the WT control (**Fig 5A**).

Consistent with the morphological changes, mating-related genes—including *MFA1*, *MFα*, *FIG1*, and *FUS1*—were induced in the *tetON-HSF1/hsf1* mutant on both YP-K and YPD-K media relative to the WT strain (**Fig 5B**). Finally, we tested the effect of *HSF1* depletion on homothallic mating. In the absence of doxycycline, the *tetON-HSF1/hsf1* mutant underwent same-sex mating with the WT partner even on the glucose-containing YPD-K medium, albeit at a lower frequency than that observed in glucose-limiting conditions (**S1 Table** and **S4 Fig**). However, in the presence of 40 μg/mL doxycycline, neither the *tetON-HSF1/hsf1* mutant nor the WT control could undergo homothallic mating on YPD-K medium. Taken together, our results indicate that down-regulation of Hsf1 promotes mating-projection formation and same-sex mating in *C*. *albicans*, bypassing the requirement of glucose starvation.

**Fig 6:**
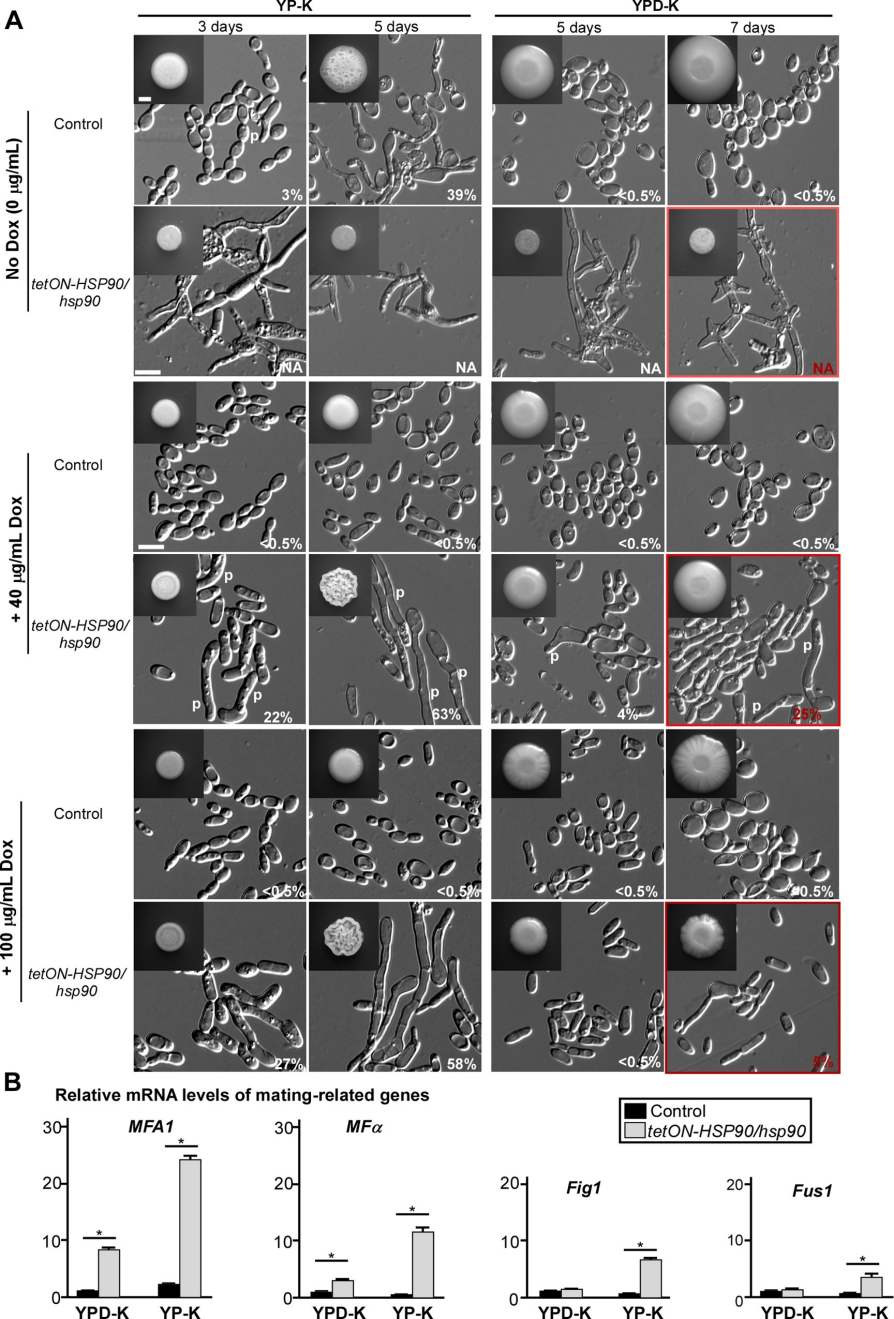
Down-regulation of Hsp90 promotes the development of mating projections. (A) Morphologies of the control and *tetON-HSP90/hsp90* mutant. Control, GH1013 + p*ACT1*-*WOR1*; *tetON-HSP90/hsp90*, a *tetON*-promoter–controlled conditional expression strain of *HSP90* with ectopically expressed *WOR1* (+ *pACT1*-*WOR1*). 1 × 10^5^ cells were spotted on YPD-K and YP-K media with or without Dox as indicated and cultured at 25°C for three, five, or seven days. Percentages of projected cells are indicated in the corresponding images. Scale bar for colonies, 2 mm (inset); scale bar for cells, 10 μm. (B) Relative expression of mating-related genes. 1 × 10^5^ cells of each strain were cultured on YPD-K medium (for seven days) or on YP-K medium (for three days) with 40 μg/mL Dox at 25°C. Relative expression levels were not tested on YP-K medium without Dox since cell viability of the *tetON-HSP90/hsp90* mutant was severely impaired. Error bars represent standard errors of technical duplicates. *p < 0.05, two-tailed Student *t* test. Experiment was repeated in a biological replicate, and a representative image is shown. The numerical data are presented in **S3 Data**. Dox, doxycycline; *FIG1*, Factor-Induced Gene 1; *FUS1*, cell FUSion 1; Hsp90, Heat shock protein 90; *MFA1*, Mating type A1; *MF*α, Mating α factor precursor; NA, not analyzed; p, mating projection; p*ACT1*, plasmid pACT1; *tetON*, tetracycline-induced; *WOR1*, White–Opaque Regulator 1; YPD-K, yeast extract-peptone-glucose-K_2_HPO_4_; YP-K, yeast extract-peptone-K_2_HPO_4_.

Next, we wanted to evaluate whether the impact of Hsf1 on homothallic mating might be mediated through its regulatory effects on *HSP90*. We constructed a *tetON-HSP90/hsp90* strain using an analogous *tetON*-promoter–controlled conditional expression strategy. As shown in **Fig 6**, culturing this strain in media containing 40 μg/mL doxycycline was required to bypass a substantial growth defect of the strain. Further, opaque cells of the conditional *tetON-HSP90/hsp90* strain were not stable, and therefore an ACTin 1 (*ACT1*) promoter controlling White–Opaque Regulator 1 (*WOR1*), the master regulator of the opaque phenotype, was introduced in the mutant to maintain the opaque state. Similar to the *tetON-HSF1/hsf1* mutant, the *tetON-HSP90/hsp90* strain underwent the development of mating projections on both YP-K and YPD-K media containing 40 μg/mL doxycycline (**Fig 6A**). However, the WT control was unable to form mating projections under the same culture conditions (**Fig 6A**). In the presence of 100 μg/mL doxycycline, the ratio of projected cells on YPD-K medium was remarkably reduced (**Fig 6A**). As expected, mating-related genes *FIG1* and *FUS1* were induced on YP-K medium, while *MFA1* and *MFα* were induced in the *tetON-HSP90/hsp90* mutant on both media relative to the WT strain in the presence of 40 μg/mL doxycycline (**Fig 6B**). As demonstrated in **S4A Fig**, the relative transcript levels of *HSF1* or *HSP90* in the *tetON-HSF1/hsf1* or *tetON-HSP90/hsP90* mutant were lower than that in the WT strain even in the presence of 40 μg/mL doxycycline. Together, these data suggest that reduction of *HSF1* or *HSP90* levels leads to the induction of a homothallic mating program and bypasses the requirement for glucose depletion.

### Transcription factors Cta4 and Cwt1 regulate the development of mating projections and same-sex mating

**Fig 7:**
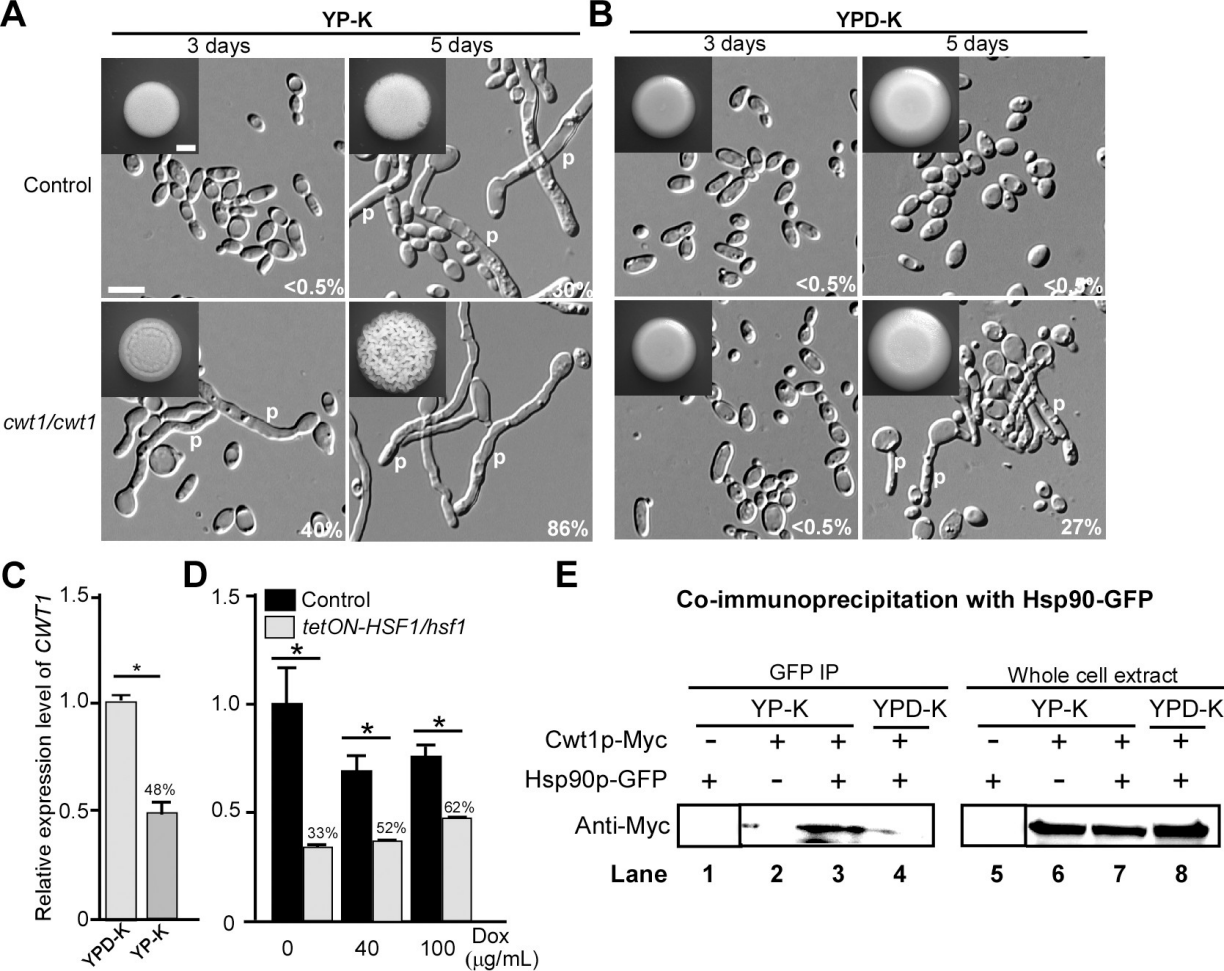
Role of the Cwt1 transcription factor in the induction of mating projections. (A and B) Morphologies of the control (GH1013 + *ARG4* + *HIS1*) and *cwt1/cwt1* mutant on YP-K (A) or YPD-K (B) medium. 1 × 10^5^ cells of each strain were spotted on YPD-K and YP-K media and cultured at 25°C for three or five days. Percentages of projected cells are indicated in the corresponding images. Scale bar for colonies, 2 mm (inset); scale bar for cells, 10 μm. (C) Relative expression levels of *CWT1* in YPD-K and YP-K media. Cells of *C*. *albicans* were spotted on YPD-K and YP-K media and cultured at 25°C for five days. (D) Relative expression levels of *CWT1* in the control (GH1013cartTA) and *tetON-HSF1/hsf1* on YP-K medium. Error bars, standard errors of technical duplicates. *p < 0.05, two-tailed Student *t* test. Percentages indicate the ratio of gene expression in *tetON-HSF1/hsf1* mutant relative to gene expression in control. The numerical data are presented in **S3 Data**. (E) Physical interaction of Cwt1 and Hsp90. Co-IP assays were performed using a strain with 13× Myc-tagged Cwt1 and GFP-tagged Hsp90. Lanes 1–4, samples co-immunoprecipitated by the GFP antibody Sepharose and analyzed by immunoblotting with the anti-Myc antibody. Lanes 5–8, whole-cell extracts analyzed by immunoblotting with the anti-Myc antibody. Experiment was performed in biological replicate, and a representative image is shown. Arg, arginine; Cwt1, Cell Wall Transcription factor 1; Dox, doxycycline; GFP, green fluorescent protein; His, histidine; Hsf1, Heat Shock transcription Factor 1; Hsp90, Heat shock protein 90; IP, immunoprecipitation; Myc, Myc epitope tag; p, mating projection; *tetON*, tetracycline-induced; *tetON-HSF1/hsf1*, *tetON*-promoter–controlled conditional expression strain of *HSF1*; YPD-K, yeast extract-peptone-glucose-K_2_HPO_4_; YP-K, yeast extract-peptone-K_2_HPO_4_.

To further characterize the regulatory mechanism of glucose starvation-induced mating, we screened a transcription factor homozygous deletion library (in an *MTL***a**/**a** background) [29] to look for mutants capable of enhanced mating projection formation. Through this functional genomic screening approach, we identified the transcription factors Cwt1 and Cta4 that function as negative and positive regulators of the development of mating projections in *C*. *albicans*, respectively (**S5 Fig** and **S6 Fig**). Homozygous deletion of *CWT1*, a gene involved in the nitrosative stress response [30], resulted in an increase in mating-projection formation on YP-K medium at three days compared to the WT control. Unlike the WT control that grew exclusively as yeast on YPD-K, *cwt1/cwt1* mutants also showed polarized growth (**S5A Fig**). As expected, mating-related genes *MFA1*, *MFα*, *FIG1*, and *FUS1* were significantly increased in the *cwt1/cwt1* mutant on YPD-K relative to a WT control (**S5B Fig**). To verify the function of Cwt1 in regulating homothallic mating, we generated an additional homozygous *CWT1* deletion mutant in the GH1013 background. Similar to the library mutant, the newly generated *cwt1/cwt1* strain exhibited a more robust development of mating projections on both YP-K and YPD-K than the WT control (**Fig 7A and 7B**), and also promoted same-sex mating on YPD-K medium (**S1 Table**). Thus, Cwt1 represses homothallic mating in *C*. *albicans*.

In contrast, deletion of *CTA4*, a previously identified genetic interactor of Hsp90 [23, 24] and a gene induced by nitric oxide, significantly repressed the development of mating projections in YP-K medium in *C*. *albicans* (**S6A Fig**). Consistently, the expression of mating-related genes was not induced in the *cta4/cta4* mutant in YP-K medium, unlike in the WT control (**S6B Fig**). However, the relative expression levels of *CWT1* were significantly increased in the *cta4/cta4* mutant, and chromatin immunoprecipitation (ChIP) assays demonstrated that Cta4 bound to the promoter regions of *CWT1* on YP-K medium (**S6C Fig**). Thus, Cta4 may provide a functional connection between Hsp90 and Cwt1 and directly regulate the transcriptional expression of *CWT1*.

Consistently, we found that the transcription level of *CWT1* was down-regulated in YP-K medium compared to the level observed in YPD-K medium (**Fig 7C**). Moreover, in the *tetON-HSF1/hsf1* background in which levels of *HSF1* were reduced relative to a WT control, we also observed a significant reduction in *CWT1* transcript levels (**Fig 7D**), suggesting that the transcriptional expression of *CWT1* is directly or indirectly regulated by Hsf1–Hsp90 signaling.

Protein sequence analysis indicated that Cwt1 contains a Zn_2_Cys_6_ motif at the carboxyl terminus and a conserved Per-Arnt-Sim (PAS) domain at the amino terminus. Since the PAS domain often interacts with Hsp90 in eukaryotic organisms [31, 32], we next tested whether Hsp90 was able to bind to Cwt1 and regulate its activity in *C*. *albicans*. As shown in **Fig 7E**, co-immunoprecipitation (IP) assays indicated that Hsp90 and Cwt1 physically interact on YP-K, but not YPD-K, medium. Therefore, Hsf1–Hsp90 signaling may regulate the activity of Cwt1 at post-transcriptional levels as well as at the transcriptional level via Cta4 in *C*. *albicans*.

### Cwt1 regulates the master regulator of a-type mating, MTLa2, to control same-sex mating

**Fig 8:**
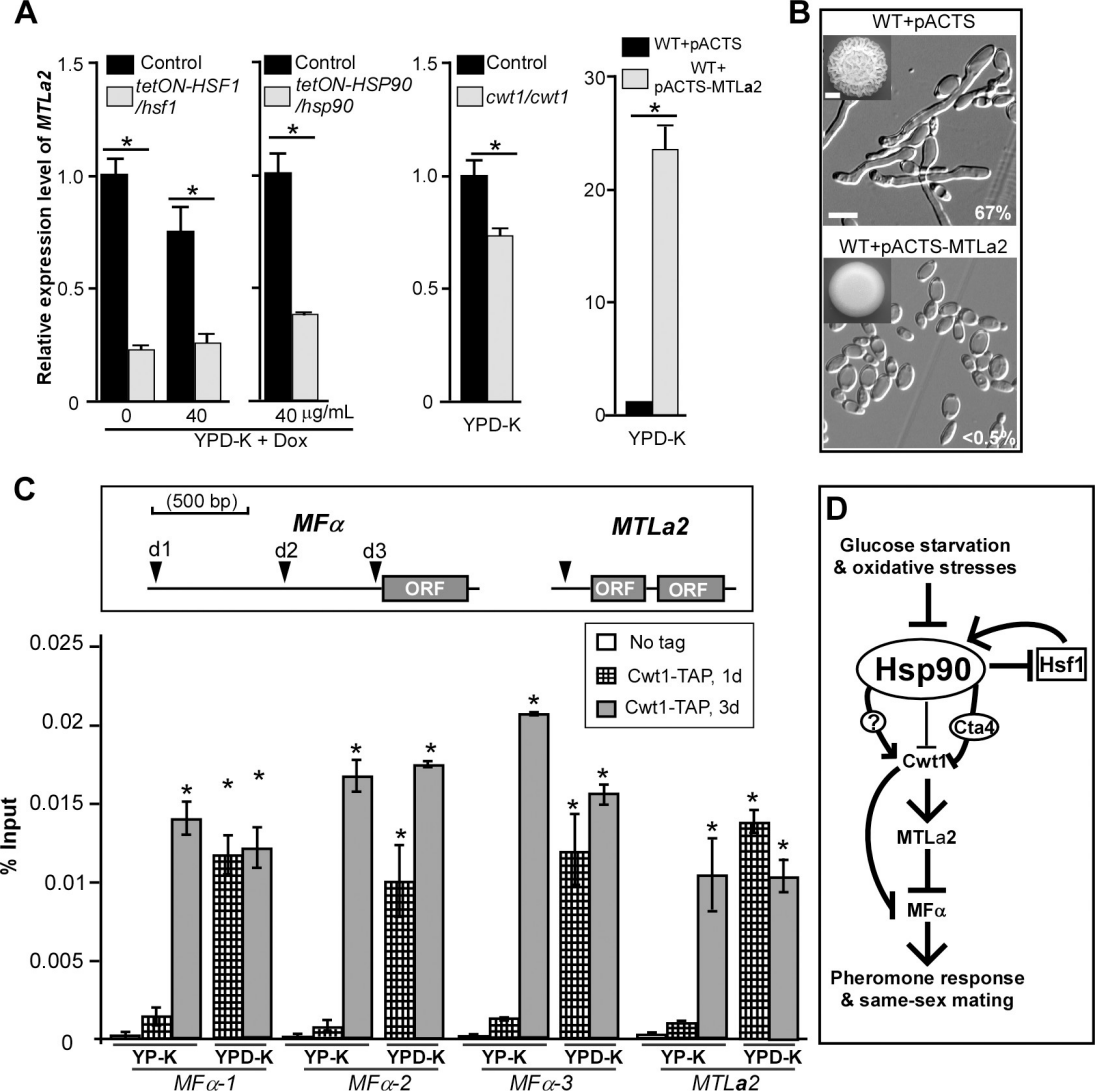
*MTLa2* regulates the development of mating projections in *C*. *albicans*. (A) Relative expression levels of *MTL****a****2* in the corresponding controls, *tetON-HSF1/hsf1*, *tetON-HSP90/hsp90*, and *cwt1/cwt1* mutants, and *MTL***a**2*-*overexpressing strain on YPD-K medium. Transcript levels were normalized to *ACT1*. Error bars, standard errors of technical duplicates. *p < 0.05, two-tailed Student *t* test. Experiment was performed in biological replicate, and a representative image is shown. (B) 1 × 10^5^ cells of each strain were spotted on YP-K medium and cultured at 25°C for five days. Morphologies of the control (WT + pACTS) and *MTL****a****2*-overexpressing strains (WT + pACTS-*MTL****a****2*) on YP-K medium. WT, GH1350a. Scale bar for colonies, 2 mm (inset); scale bar for cells, 10 μm. (C) Cwt1 binds to the promoters of *MTL****a****2* and *MFα*. ChIP assays were performed in TAP-tagged Cwt1 strains. Percentages of input genomic DNA are indicated. 1 × 10^5^ cells of each strain were spotted on YP-K or YPD-K media and cultured at 25°C for one or three days. Dark arrows indicate detected promoter regions. d1, d2, and d3, three detected sites of MFα. Error bars represent standard error of two technical replicates. *p < 0.05, two-tailed Student *t* test. Experiment was performed in biological replicate with a representative image shown. The data for the untagged control correspond to samples harvested at day 1. The numerical data are presented in **S3 Data**. (D) Regulatory model of glucose-starvation–or oxidative-stress–induced same-sex mating in *C*. *albicans*. Glucose starvation or oxidative stresses cause the overwhelming of the Hsf1/Hsp90 functional capacity that regulates the transcriptional expression and activity of *CWT1* in both direct and indirect manners. Cwt1 regulates same-sex mating through the control of *MF*α or *MTL***a**2. *ACT1*, ACTin 1; ChIP, chromatin immunoprecipitation; Cta4, *Candida* TransActivating protein 4; Cwt1, Cell Wall Transcription factor 1; Dox, doxycycline; Hsf1, Heat Shock transcription Factor 1; Hsp90, Heat shock protein 90; *MF*α, Mating α factor precursor; *MTL*, Mating type locus; pACTS, plasmid pACTS; TAP, Tandem affinity purification; *tetON*, tetracycline-induced; *tetON-HSF1/hsf1*, *tetON*-promoter–controlled conditional expression strain of *HSF1*; WT, wild type; YPD-K, yeast extract-peptone-glucose-K_2_HPO_4_; YP-K, yeast extract-peptone-K_2_HPO_4_.

MTL**a**2 is required for the maintenance of **a**-cell identity [33]. Inactivation of MTL**a**2 induces the expression of α-pheromone in *MTL***a**/**a** cells of *C*. *albicans* [33]. Given our observation that glucose starvation induced the expression of both pheromone precursors *MFA1* and *MFα*, we assessed whether homothallic mating induced in glucose-limiting conditions was governed by changes in *MTL****a****2* levels. To test this, we overexpressed *MTL****a****2* and observed a suppression in the development of mating projections under glucose-limiting conditions (**Fig 8A and 8B**). Next, to test whether compromise of Hsf1–Hsp90–Cwt1 signaling impaired *MTL****a****2* expression, we monitored *MTL****a****2* levels in our *tetON-HSF1/hsf1* and *tetON-HSP90/hsp90* mutants. Down-regulation of *HSF1* or *HSP90* or deletion of *CWT1* led to significantly decreased expression of *MTL****a****2* on YPD-K medium (**Fig 8A**), implicating this stress-response signaling in the regulation of *MTL****a****2* expression. It has been indicated that Cwt1 has potential binding sites in the promoter regions of *MTL****a****2* and *MFα* genes [34]. Using ChIP assays, we observed that Cwt1 directly binds to the promoter regions of both *MTL****a****2* and *MFα* on YPD-K medium in the one-day cultures, and this binding activity was observed on both on YP-K and YPD-K media in the three-day cultures (**Fig 8C**). These results suggest that Cwt1 directly regulates mating-related gene expression.

**Fig 9:**
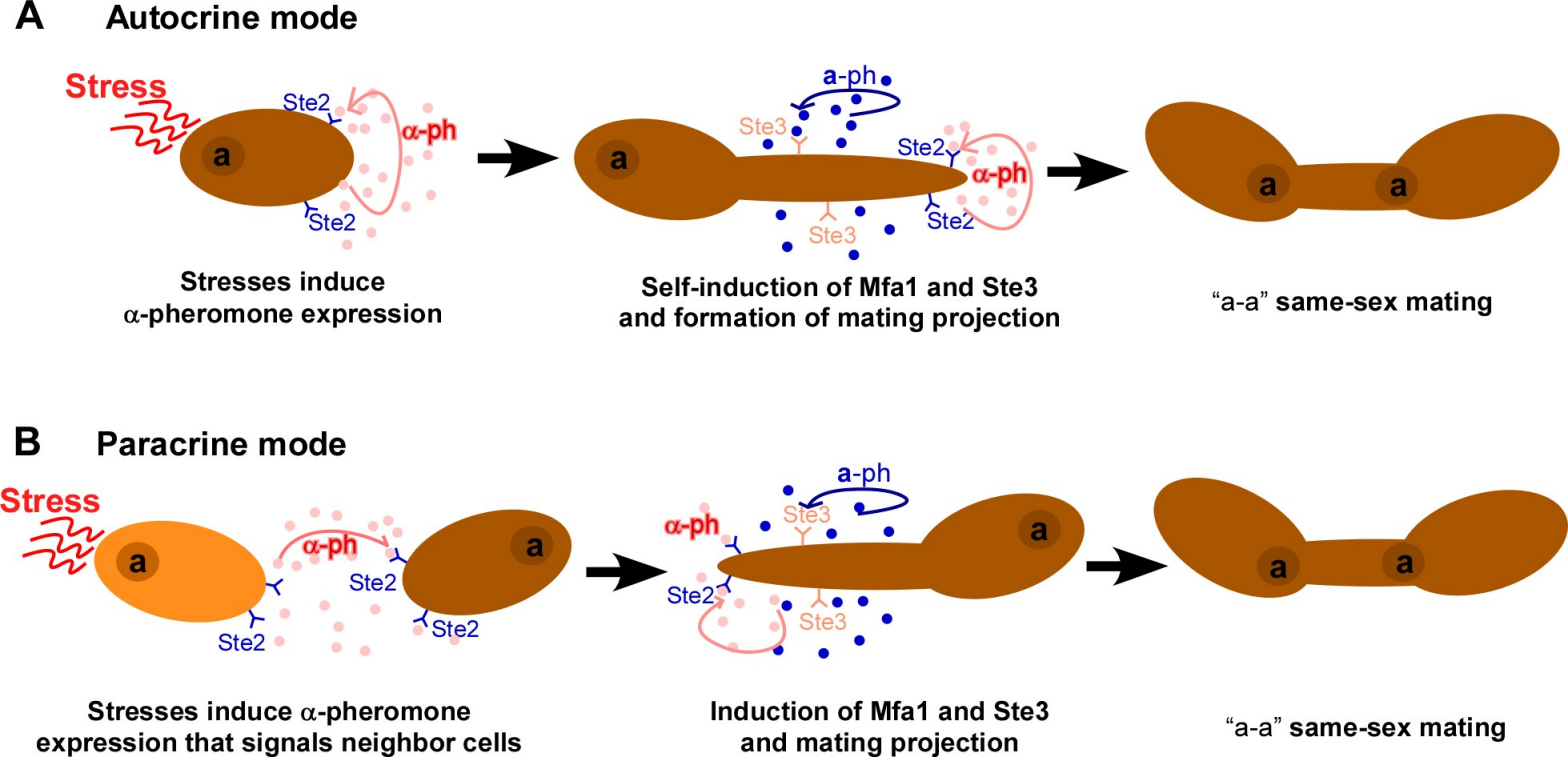
**Potential autocrine (A) and paracrine (B) pheromone response models for stress-induced mating-projection formation and same-sex mating.** Neither α-pheromone nor **a**-pheromone is constitutively expressed in “**a**” cells of *C*. *albicans*. Glucose starvation or oxidative stress first induces α-pheromone and its receptor Ste2 expression in “**a**” cells. α-pheromone binds to Ste2 and activates the pheromone-response pathway, which subsequently induces the expression of **a**-pheromone. “**a**” cells then become mating competent as both “**a**” and “α” types. The activation of the pheromone-response pathway promotes mating projection formation and same-sex mating. Autocrine pheromone response, self-activation (A); paracrine pheromone response, activation of neighbor cells (B). *MFA1*, Mating type A1; *STE2*, STErile 2.

Overall, our data suggest a model in which Hsf1–Hsp90 signaling controls the expression of Cwt1 through Hsp90 genetic interactors such as Cta4 as well as the activity of Cwt1 post-translationally through a physical interaction with Hsp90. Cwt1 is a dimeric Zn_2_Cys_6_ zinc-finger transcription factor. The physical interaction between Hsp90 and Cwt1 could inhibit the dimerization of Cwt1 through the PAS domain in *C*. *albicans*, as observed in other eukaryotic organisms [32]. Cwt1 binds to the promoters of *MF*α or *MTL***a**2 and regulates their transcriptional expression (**Fig 8D**). It has been demonstrated that same-sex mating in *C*. *albicans* is induced by the autocrine and/or paracrine pheromone response [16]. Therefore, the activated pheromone signaling by the environmental cues could then promote the development of mating projections and same-sex mating, possibly through these two response modes (**Fig 9**).

## Discussion

The predominantly clonal nature of *C*. *albicans* greatly limits the occurrence of heterothallic mating between two competent cells with opposite mating types in its natural environment. It would be difficult for a mating-competent cell of *C*. *albicans* to find a competent cell with an opposite mating type to mate. Although the efficiency of same-sex mating is much lower than that of opposite-sex mating under laboratory conditions in *C*. *albicans* [16], same-sex mating involving cells of a single type would lower the barrier to finding a suitable partner [35]. However, the requirement of α-pheromone production in close proximity to drive homothallic mating between two *MTL****a*** cells or the need to inactivate the Bar1 protease challenges the relevance of same-sex mating in nature [16]. This is especially true given that neither natural *C*. *albicans* mutants with loss of Bar1 function nor environmental conditions suppressing Bar1 activity have been discovered. Thus, the question remains: can *C*. *albicans* undergo frequent same-sex mating in natural environments?

In our current study, we find that glucose starvation and oxidative stress induce same-sex mating in *MTL****a*** cells of *C*. *albicans*, providing an environmentally relevant means to drive sexual reproduction in this species. *C*. *albicans* is commonly exposed to glucose starvation because glucose is limited in its natural niches such as the mouth, lower gut, and environments contaminated with human or animal excreta. However, under the same conditions, we did not observe same-sex mating in opaque Mating type α (*MAT*α) cells, perhaps because of *MTL***a**2, a key regulator in this induction in *MTL***a** cells. *MAT*α/α cells may use a different environmental cue for the induction of same-sex mating. Similar to reports highlighting that the fungal pathogen *Cryptococcus neoformans* undergoes mating on pigeon guano [36], we found that animal feces promoted same-sex mating in *C*. *albicans* (**S3 Fig**). Given that the gut of human or warm-blooded animals is a major natural niche for *C*. *albicans* [12], animal feces would be a major source for most nutritional components. Furthermore, growth on *C*. *albicans* debris medium containing no additional nutrients also induced same-sex mating (**S3 Fig**). When yeast grows on agar-only medium, a portion of cells undergo cell death and release nutrients for the growth and survival of neighboring cells [37]. Under these glucose-limiting conditions, the frequency of same-sex mating in *C*. *albicans* ranged from 1 × 10^−7^ to 3 × 10^−5^ (**S3 Fig**), suggesting that the average-aged colony (containing approximately 1 × 10^8^ cells) can produce tens to hundreds of mating progeny. Therefore, the occurrence of same-sex mating under glucose starvation conditions could be considerably more frequent in nature than was originally thought.

The notion that environmental stress may serve as a trigger to induce more frequent sexual reproduction in *C*. *albicans* has been previously proposed based on a series of observations [38]. Poor nutritional conditions increase the frequency of opposite-sex mating [39]. Oxidative stress promotes the induction of the white-to-opaque switch [40], and recombination occurs more frequently following exposure to several types of stress, which would provide a critical step for the homozygosis of the *MTL* locus. Despite these lines of evidence, our work provides the seminal example of stressful conditions governing homothallic mating in *C*. *albicans*. In *C*. *neoformans*, sexual mating is stimulated by stresses such as nitrogen starvation, desiccation, and darkness [41]. Moreover, treatment of *S*. *pombe* with the oxidative agent H_2_O_2_ promotes the generation of meiotic spores [42]. Therefore, exposure to harsh environmental conditions could be a general signal for diverse fungal species to integrate environmental response pathways with increased sexual reproduction.

The evolutionarily conserved regulators Hsf1 and Hsp90 play a primary and global role in orchestrating stress responses in eukaryotic organisms [43]. Glucose starvation results in the production of ROSs (**Fig 3**) that would likely cause protein damage in *C*. *albicans*. Under these situations, the functional capacity of Hsp90 could be overwhelmed, causing it to be titrated away from its basal client proteins while it deals with more global problems of protein misfolding. Consistently, a number of heat-shock-protein–encoding genes, including *HSP90*, were up-regulated in response to glucose deprivation (**Fig 4** and **S1 Data**).

In our study, we implicate the Cta4 and Cwt1 transcription factors, which regulate the nitrosative or nitric oxide stress response [30], as downstream effectors of the Hsf1–Hsp90 signaling and mating-response pathways (**Fig 8**). Cta4 has been previously reported as a genetic interactor of Hsp90 [23, 24] and represses the transcriptional expression of *CWT1*. Moreover, we demonstrate that Hsp90 directly binds to Cwt1 on YP-K but not YPD-K medium, perhaps through the conserved PAS motif of Cwt1. To our knowledge, this is the first time that Hsf1 or Hsp90 have been implicated in sexual mating in fungi, and our results suggest that diverse cellular stresses capable of overwhelming the function of these regulators, including elevated temperature, may also promote sexual reproduction in *C*. *albicans*.

Inactivation of the Bar1 protease or the Dipeptidyl aminopeptidase YC1 (Yci1) domain protein Opaque Formation Regulator 1 (Ofr1) has been shown to induce same-sex mating in *C*. *albicans* [16, 44]. We observed that *BAR1* was not down-regulated but rather highly induced upon glucose starvation (**Fig 1**). Further, inactivation of Ofr1 allows *MTL***a**/α cells to undergo same-sex mating as the “**a**” mating type [44]. Although glucose starvation can only induce same-sex mating in *MTL***a/a** cells but not in *MTL***a**/α or *MTL*α/α cells, other unidentified environmental conditions may allow *MTL***a**/α or *MTL*α/α cells to undergo homothallic mating. Therefore, glucose-starvation–induced same-sex mating appears to be independent of Bar1 and Ofr1.

In summary, we uncover a novel, to our knowledge, environmental trigger, glucose depletion, capable of acting as a signal for sexual mating in *C*. *albicans*, which not only sheds light on the biology of this pathogen but also expands the diverse repertoire of sexual reproduction modes in fungi. This strategy is different from that used by *S*. *cerevisiae*, *S*. *pombe*, and *C*. *neoformans*, despite the fact that the evolutionarily diverse yeast species achieve a same output for homothallism. Unisexual reproduction generates aneuploidy and de novo phenotypic diversity in fungi [45, 46], thus providing a selective advantage under stressful conditions over those organisms propagating exclusively in an asexual manner. *C*. *albicans* exists as an obligate diploid in nature, with many heteryozygous loci between homologous chromosomes. Therefore, when tetraploid intermediate cells generated from same-sex mating return to a lower ploidy state, there is substantial opportunity for the generation of genetically diverse progeny. These tetraploid strains could serve as a capacitor for generating distinct aneuploidies and randomly combined chromosome sets in order to facilitate the evolution of new traits to adapt to changing environments.

## Materials and methods

### Strains and culture conditions

The strains used in this study are listed in supplementary **S2 Table**. YPD (20 g/L peptone, 10 g/L yeast extract, 20 g/L glucose) and modified Lee’s glucose medium supplemented with 5 μg/ml phloxine B [47] were used for routine growth of *C*. *albicans*. Solid YPD-K (20 g/L peptone, 10 g/L yeast extract, 20 g/L glucose, 2.5 g/L K_2_HPO_4_, 20 g/L agar) and YP-K (20 g/L peptone, 10 g/L yeast extract, 2.5 g/L K_2_HPO_4_, 20 g/L agar) media were used for mating-projection induction assays. Peptone, yeast extract, and agar were purchased from BD Biosciences (BD Bacto, Cat. Nos., 211677, 212750, G8270, and 214010; BD Biosciences, Sparks, MD, USA). Glucose, phloxine B, and K_2_HPO_4_ were purchased from Sigma-Aldrich (Cat. Nos., G8270, P2759, and P9666; Sigma-Aldrich, St. Louis, MO, USA). The pH of YPD-K and YP-K media were about 7.3. Opaque cells were used for all mating and mating projection formation assays.

Sorbitol medium was made according to a previous study [48]. YPD-K, YP-K, agar-only (3%), agar + *C*. *albicans* debris, or agar + 3% mouse feces media were used for quantitative mating assays. To make the *C*. *albicans* debris medium, approximately 1 × 10^10^ cells of SC5314 were collected from an overnight YPD culture, washed with ddH_2_O, frozen at −80°C, and ground with glass beads. Cell debris and 2% agar were mixed and resuspended in 100 mL ddH_2_O for autoclaving. To make the mouse feces medium, 2% agar and 3% mouse feces (w/v) were mixed and resuspended in ddH_2_O. Before subjecting to autoclaving, 0.25% K_2_HPO_4_ was added to the agar-only (3%), agar + *C*. *albicans* debris, or agar + 3% mouse feces media for pH buffering. Synthetic complete medium (SCD) lacking corresponding nutrients (uridine, histidine [His], and/or arginine [Arg]) was used for selectable growth in quantitative mating assays.

To make H_2_O_2_-containing YPD-K medium, 200 μl of 5 mM H_2_O_2_ was spread onto YPD-K medium plates. Opaque cells of GH1350a were first grown on Lee’s glucose medium at 25°C for three days. 1 × 10^6^ cells in 10 μL of ddH_2_O were spotted onto different H_2_O_2_-containing YPD-K media and cultured at 25°C for three to five days.

### Construction of plasmids

To inactivate the *Sac*II site of *tetON*-promoter–containing plasmid pNIM1 [49], the plasmid was first digested with *Sac*II and then filled in with the Klenow fragment of DNA polymerase I. The blunt ends were ligated to generate the *Sac*II-free plasmid pNIMsx. To construct the plasmids pNIMsx-HSP90con and pNIMsx-HSF1con for conditional knockout of *HSP90* and *HSF1*, respectively, one fragment of partial ORF region of *HSP90* or *HSF1* (with *Sal*I and *Sac*II sites) and another fragment of the corresponding 5′-UTR region (with *Sac*II and *Bgl*II sites) were simultaneously subcloned into the *Sal*I and *Bgl*II sites and replaced the GFP cassette.

To construct the nourseothricin-resistant plasmid pACTS, a ca*SAT1* fragment was amplified from pNIM1 [49] and subcloned into the *Hind*III*/Kpn*I site of pACT1 [50]. A fragment containing the *MTL****a****2* ORF region was then subcloned into the *Eco*RV/*Hind*III site of pACTS, generating the overexpressing plasmid pACTS-MTL**a**2.

To create a Myc-tagged Cwt1 plasmid, the *CdHIS1* cassette was amplified from plasmid pSN52 and inserted into plasmid pACT1 at the *Cla*I site, generating plasmid pACT1-HIS1. A fusion PCR product containing the *CWT1* ORF region and a C-terminal 13× Myc tag were prepared and subcloned into the *Eco*RV/*Kpn*I site of pACT1-HIS1, yielding plasmid pACT1-CWT1-Myc-HIS1.

A *TAP-ARG4* cassette flanked by approximately 70-bp-5′– and 3′–homologous sequences of *CWT1* was amplified from strain CaLC2993 with primer pair LT1266 and LT1267 and was then transformed into the WT strain SN95 (CaLC239) to create TAP-tagged strain LTS1036 [51, 52]. The fragment containing the *CWT1* ORF region and a C-terminal TAP-ARG4 tag was amplified from strain LTS1036 with oligonucleotides LT1271/LT1272 and then subcloned into the *Eco*RV/*Kpn*I site of pACT1 [50], generating plasmid pACT-CWT1-TAP-ARG4.

A *TAP-ARG4* cassette flanked by approximately 70-bp-5′– and 3′–homologous sequences of *CTA4* was amplified from strain CaLC2993 with primer pair LT1459 and LT1460 and was then transformed into the WT strain SN95 (CaLC239) to create TAP-tagged strain LTS1071 [52]. The fragment containing the *CTA4* ORF region and a C-terminal *TAP-ARG4* tag were amplified from strain LTS1071 with oligonucleotides LT1271/LT1462 and then subcloned into the *Eco*RV/*Kpn*I site of pACT1, generating plasmid pACT-CTA4-TAP-ARG4.

### Construction of *C*. *albicans* strains

To construct the conditional knockout mutants of *HSP90* in strain GH1013, we first replaced the promoter of one allele with the *tetON* promoter using *Sac*II-digested plasmid pNIMsx-HSP90con, generating mutant *tetON-HSP90/HSP90*. The other allele of *HSP90* was then replaced with the *ARG4* cassette amplified from pRS-ARG4ΔSpeI [53] with oligonucleotides HSP90-5DR/HSP90-3DR, generating the conditional mutant *tetON-HSP90/hsp90*. Since opaque cells of the *tetON-HSP90/hsp90* mutant were not stable, the master regulator *WOR1* was overexpressed in this mutant using plasmid pACT1-WOR1 [50]. To construct the conditional knockout mutant of *HSF1*, we deleted the first allele in strain GH1013 using the *URA3* cassette amplified from pGEM-URA3 [53] with oligonucleotides HSF1-5DR/HSF1-3DR, generating the mutant *hsf1*::*URA3/HSF1*. Then, we replaced the promoter region of the second allele of *HSF1* with the *tetON* promoter using *Sac*II-digested plasmid pNIMsx-HSF1con, generating the mutant *tetON-HSF1/hsf1*. The *cartTA* cassette (reverse *tet* repressor) is under the control of the white-cell–specific *ADH1* promoter in the plasmid pNIM1 [49]. To increase the expression level of *cartTA* in opaque cells, the cassette was integrated into the opaque-specific *OP4* locus in the *tetON-HSF1/hsf1* and *tetON-HSP90/hsp90* mutants by transformation with fusion PCR products of *cartTA-ARG4*. pNIM1 and pRS-ARG4ΔSpeI were used as the primary template.

To delete both alleles of *CWT1*, the *HIS1* and *ARG4* markers flanked by *CWT1* gene 5′- and 3′-fragments were amplified with fusion PCR assays and sequentially transformed into strain GH1013 as described previously [53]. The plasmids pGEM-HIS1 and pRS-ARG4ΔSpeI were used as the PCR templates. All oligonucleotides used are listed in **S3 Table**.

To determine Cwt1-binding targets, a TAP-tagged Cwt1-ecotopic strain was constructed. The plasmid pACT-CWT1-TAP-ARG4 was linearized with *Asc*I and transformed into strain SN95 [54] to create a TAP-tagged Cwt1-overexpressing strain (LTS1039). The function of TAP-tagged Cwt1 was verified by mating projection formation assays. To construct the *MTL**a**2*-overexpressing strain, plasmid pACTS-MTL**a**2 was linearized with *Asc*I and transformed into strain GH1350**a**.

To construct a TAP-tagged Cta4-ecotopic expression strain, the plasmid pACT-CTA4-TAP-ARG4 was linearized with *Asc*I and transformed into strain SN95 to create TAP-tagged CTA4-ecotopic expression strain (LTS1079). To construct a GFP-tagged Hsp90 strain, a *HSP90-GFP-SAT1* fusion fragment into the pACT1 plasmid [50], generating plasmid pACT1-HSP90-GFP-SAT1. The plasmids pACT1-HSP90-GFP-SAT1 and pACT1-CWT1-Myc-HIS1 were linearized with *Asc*I and subsequently transformed into strain SN95 [54], yielding strain LTS1062 with a GFP-tagged HSP90 and 13× Myc-tagged *CWT1* allele.

### Construction of GFP-reporter strains

To construct the MF**a**1p-GFP, MFαp-GFP, FIG1p-GFP, and FUS1p-GFP reporter strains, GH1013 was transformed with PCR products of the *GFP-caSAT1* cassette amplified from plasmid pNIM1 with corresponding primers. The forward and reverse primers contain a 60-bp flanking sequence homologous to the promoter and 3′-UTR regions of *MF****a****1*, *MFα*, *FIG1*, or *FUS1*, respectively. Correct integration of the transformations was verified with PCR assays.

### Quantitative mating assay

Same-sex mating assays were performed according to our previous publications with slight modifications [55]. Briefly, opaque cells of two “**a**” strains (1 × 10^7^ for each) were mixed, spotted onto different media, and cultured at 25°C for three to seven days as indicated in the main text. Mating mixtures were then replated onto SCD-His, -Arg, -uridine, -His-Arg, or -Arg-uridine dropout media for prototrophic selection growth. Colonies grown out on the three types of plates were counted, and mating efficiency was calculated.

### Flow cytometry analysis

*C*. *albicans* cells were incubated in liquid SCD medium with shaking at 30°C overnight, harvested, washed, and resuspended in 1× TE buffer (10 mM Tris, 1 mM EDTA [pH 8.0]). Cells were then fixed with 70% ethanol for two hours at room temperature and washed with 1× TE buffer before treating with 1 mg/mL RNase A and 5 mg/ml proteinase K. Propidium iodide (PI, 25 μg/ml) staining assays were then performed. Stained cells were washed and resuspended in 1× TE buffer for DNA content analysis. A total of approximately 30,000 cells of each sample were used for flow cytometry assays, and the results were analyzed using software FlowJo 7.6.1.

### Quantitative real-time PCR and RNA-Seq assays

Quantitative real-time PCR assays were performed according to our previous publications with modifications [56]. Cells were collected from cultures grown on solid plates as described in the main text. One μg of total RNA per sample was used to synthesize cDNA with RevertAid H Minus Reverse Transcriptase (Thermo Scientific, Waltham, MA, USA). Quantification of transcripts was performed in Bio-Rad CFX96 real-time PCR detection system using SYBR green. The signal from each experimental sample was normalized to expression of the *ACT1* gene.

For RNA-Seq assays, opaque cells of *C*. *albicans* were grown to stationary phase on Lee’s glucose medium at 25°C for five days and then spotted onto YPD-K and YP-K medium at 25°C for 60 hours of incubation. Two biological repeats were performed for each condition. Cells were harvested, and total RNA was extracted. RNA-Seq analysis was performed by the company Berry Genomics (Beijing, China) as described previously [57]. Briefly, approximately 10 million (M) reads were sequenced in each library of the samples. The library products were then sequenced using an Illumina HiSeq 2500 V4 (Illumina, San Diego, CA, USA). Illumina software OLB_1.9.4 was used for base calling. The raw reads were filtered by removing the adapter and low-quality reads (the percentage of low-quality bases with a quality value ≤3 was >50% in a read). Clean reads were mapped to the genome of *C*. *albicans* SC5314 using TopHat (version 2.1.1) and Cufflinks (version 2.2.1) software [58]. Relative gene expression levels were calculated using the fragments per kb per million reads (FPKM) method. To be considered significantly differentially expressed, a gene must satisfy three criteria: (1) an FPKM value higher than or equal to 20 at least in one sample, (2) a fold change value higher than or equal to 1.5 (except for the Functional categories sheet of **S1 Data**, in which a 2-fold change cutoff was used), and (3) an adjusted *p*-value (false discovery rate [FDR]) lower than 0.05.

### Chromatin IP and co-IP assays

ChIP assays were performed as described previously [52, 59]. Briefly, untagged (CaLC239, SN95) and pACT1-Cwt1-TAP–tagged (LTS1039) *C*. *albicans* strains were spotted on YP-K medium and incubated at 25°C for one and three days. Cells were collected and fixed in 1× PBS containing 1% formaldehyde and incubated with gentle rocking for 20 min at room temperature. The crosslinking reaction was quenched by adding 2.5 M glycine to a final concentration of 125 mM, and cells were mixed for 5 min at room temperature. Cells were harvested, washed with 1× PBS, and homogenized in ice-cold lysis buffer using a bead beater. Sonication was performed with a Diagenode Bioruptor (Diagenode, Denville, NJ, USA) (12 min, high setting, 30 s on, 1 min off) to obtain chromatin fragments of an average size of 250–1,000 bp. The chromatin was immunoprecipitated with 50 μl packed IgG Sepharose 6 Fast Flow matrix (GE Healthcare, Chicago, IL, USA). The Sepharose matrix was washed, and immunoprecipitated chromatin DNA was eluted and de-crosslinked at 65°C overnight. Quantitative real-time PCR assays were performed to determine Cwt1 targets.

Cells grown on YP-K and YPD-K media were harvested and suspended in lysis buffer containing 50 mM Na-HEPES (pH 7.5), 450 Mm NaOAc (pH 7.5), 1 mM EDTA, 1 mM EGTA, 5 Mm MgOAc, 5% glycerol, 0.25% NP-40, 3 mM DTT, 1 mM PMSF, and EDTA-free protease inhibitor mix (Cat. No.,11873580001; Roche Diagnostics, Mannheim, Germany). Cells were lysed using a beadbeating instrument by five rounds of beating (50 s beating plus 1 min cooling process on ice for each round). The supernatant of cell lysates was collected and incubated with Sepharose beads conjugated anti-GFP monoclonal antibody at 4°C for three hours. The beads were washed for four times with the lysis buffer and then boiled for 10 min in 1× sodium dodecyl sulfate (SDS) sample buffer. Immunoprecipitated proteins were separated through an SDS-10% polyacrylamide gel and used for western blotting analysis using anti-Myc monoclonal antibodies (Cat. No., OP10; MilliporeSigma, Billerica, MA, USA).

### Intracellular ROS determination

Cells were cultured for one to five days on YP-K or YPD-K medium at 25°C. Cells were then harvested and washed in 1 × PBS and incubated with DCFDA (Beyotime, Shanghai, China) for 30 min at 37°C. After washing, fluorescence intensity reflecting the ROS level was measured at 488 nm using ELISA and was normalized according to the cell numbers.

## Supporting information

S1 Fig Development of polarized growth under different glucose concentrations. 1 × 10^7^ cells of strain GH1350a were spotted on media containing different levels of glucose and cultured at 25°C for three or seven days. Percentages of projected cells are indicated in the corresponding images. The percentage of projected cells decreases with the increase of glucose level.
(TIF)

S2 Fig Expression of mating-related genes in GFP-reporter strains under glucose starvation conditions. 1 × 10^5^ cells of each GFP-tagged strain (GH1013 background) were spotted on YP-K or YPD-K medium and cultured at 25°C for five days. Scale bar, 10 μm. DIC, differential interference contrast; GFP, green fluorescent protein; YPD-K, yeast extract-peptone-glucose-K_2_HPO_4_; YP-K, yeast extract-peptone-K_2_HPO4.
(TIF)

S3 Fig Same-sex mating of *C. albicans* under conditions mimicking natural environments. (A) Diagram of experimental procedures. (B, C, and D) Mating efficiency on 3% agar without additional nutrients (B), on agar containing 3% mouse feces (C), and agar containing *C*. *albicans* debris (D). 1 × 10^7^ cells of GH1013 and 1 × 10^7^ cells of GH1350a were mixed and cultured on different medium plates at 25°C for three to seven days. Mating mixtures were replated onto SCD-Arg, SCD-His, and both dropout plates for selectable growth and mating efficiency calculation. For mating on agar without additional nutrients (B), a portion of cells underwent cell death and released nutrients for the survived cells. (E) Mating on sorbitol medium (opaque filamentation inducing medium). The numerical data are presented in **S3 Data**. Arg, arginine; His, histidine; SCD, synthetic complete medium.
(TIF)

S4 Fig Relative expression levels of *HSF1* and *HSP90* and FACS analysis of mating progeny. (A) Relative transcriptional expression levels of *HSF1* or *HSP90* in the control and *tetON-HSF1/hsf1* or *tetON-HSP90/hsp90* mutant on YP-K and YPD-K media with or without doxycycline (40 μg/mL). 1 × 10^5^ cells were spotted on YP-K or YPD-K medium and cultured at 25°C for three days. Error bars, standard errors of technical duplicates *p < 0.05, two-tailed Student *t* test. Experiment was performed in biological replicate and representative image is shown. (B) FACS analysis of the DNA content of progeny strains. Parental strain GH1350a used as a diploid control. Mating progeny contain DNA content corresponding to 4C and 8C peaks confirming their tetraploid nature. This figure is related to the quantitative results presented in supplementary S1 Table. The numerical data are presented in **S3 Data**. FACS, Fluorescence-activated cell sorting; Hsf1, Heat Shock transcription Factor 1; Hsp90, Heat shock protein 90; *tetON*, tetracycline-induced; *tetON-HSF1/hsf1*, *tetON*-promoter–controlled conditional expression strain of *HSF1*; YPD-K, yeast extract-peptone-glucose-K_2_HPO_4_; YP-K, yeast extract-peptone-K_2_HPO4.
(TIF)

S5 Fig Identification of the *cwt1/cwt1* mutant by screening a transcription factor mutant library of *C. albicans*. (A) Formation mating projections in the *cwt1/cwt1* mutant on YPD-K and YP-K media. 1 × 10^5^ cells of each strain were spotted on different media and cultured at 25°C for three or five days. Scale bar for colonies, 2 mm; scale bar for cells, 10 μm. (B) Relative expression levels of mating-related genes in the control (GH1350a) and *cwt1/cwt1* mutant on YPD-K and YP-K media. Error bars, standard errors. *p < 0.05, two-tailed Student *t* test. Two biological and two technical repeats were performed, respectively. The numerical data are presented in **S3 Data**. Cwt1, Cell Wall Transcription factor 1; p, mating projection; YPD-K, yeast extract-peptone-glucose-K_2_HPO_4_; YP-K, yeast extract-peptone-K_2_HPO4.
(TIF)

S6 Fig Identification of the *cta4/cta4* mutant by screening a transcription factor mutant library of *C. albicans*. (A) Colony and cellular morphologies of the control (WT, GH1350a) and *cta4/cta4* mutant on YP-K medium. 1 × 10^5^ cells of each strain were spotted on different media and cultured at 25°C for five days. Scale bar for colonies, 2 mm; scale bar for cells, 10 μm. (B) Relative expression levels of *CWT1* and mating-related genes in the control (GH1350a) and *cta4/cta4* mutant on YPD-K and YP-K media. Cells of *C*. *albicans* used for qRT-PCR assays were cultured at 25°C for five days. Error bars, standard errors. *p < 0.05, two-tailed Student *t* test. Two biological and two technical repeats were performed, respectively. (C) Cta4 binds to the promoters of *CWT1*. ChIP assays were performed in TAP-tagged Cta4 strains. Cells of *C*. *albicans* used for ChIP assays were grown on YP-K or YPD-K medium at 25°C for 24 hours. Percentages of input genomic DNA are indicated. Dark arrows indicate detected promoter regions. d1, d2, and d3, three detected sites of *CWT1*. Error bars represent standard error of two technical replicates. *p < 0.05, two-tailed Student *t* test. Experiment was performed in biological replicate with a representative image shown. The numerical data are presented in **S3 Data**. ChIP, chromatin immunoprecipitation; Cta4, *Candida* TransActivating protein 4; Cwt1, Cell Wall Transcription factor 1; p, mating projection; qRT-PCR, quantitative reverse transcription PCR; WT, wild type; YPD-K, yeast extract-peptone-glucose-K_2_HPO_4_; YP-K, yeast extract-peptone-K_2_HPO4.
(TIF)

S1 Table Efficiency of same-sex mating in the *tetON-HSF1/hsf1, tetON-HSP90/hsp90* and *cwt1/cwt1* mutants. Cwt1, Cell Wall Transcription factor 1; Hsf1, Heat Shock transcription Factor 1; Hsp90, Heat shock protein 90; *tetON*, tetracycline-induced; *tetON-HSF1/hsf1*, *tetON*-promoter–controlled conditional expression strain of *HSF1*.
(DOC)

S2 Table Strains used in this study. (XLSX)

S3 Table Primers used in this study. (XLS)

S1 Data RNA-Seq dataset. RNA-Seq, RNA sequencing
(XLSX)

S2 Data *HSP90* genetic interactors that differentially expressed in YP-K and YPD-K media. Hsp90, Heat shock protein 90; YPD-K, yeast extract-peptone-glucose-K_2_HPO_4_; YP-K, yeast extract-peptone-K_2_HPO_4_
(XLSX)

S3 Data Excel files containing the underlying numerical data for Figs 1C, 3A, 3C, 3D, 5B, 6B, 7C, 7D, 8AC, S3B, S3C, S3D, S3E, S4A, S5B, S6B and S6C. (XLSX)

## Abbreviations

ACT1: ACTin 1
AIF1: Apoptosis-Inducing Factor 1
Arg: arginine
Bar1: BARrier 1
CEK: Candida ERK-family protein kinase
ChIP: chromatin immunoprecipitation
Cta4: *Candida* TransActivating protein 4
Cwt1: Cell Wall Transcription factor 1
DIC: differential interference contrast
Dox: doxycycline
ERK: ERK-family protein kinase
FACS: Fluorescence-activated cell sorting
FDR: False Discovery Rate
FIG1: Factor-Induced Gene 1
FPKM: fragments per kb per million reads
FUS1: cell FUSion 1
GFP: green fluorescent protein
GPI: glycosylphosphatidylinisotol
GST3: Glutathione S-transferase 3
His: histidine
HMX1: HeMe oXygenase
HO: HOmothallic switching
Hsf1: Heat Shock transcription Factor 1
Hsp90: Heat shock protein
IP: immunoprecipitation
KAR2: KARyogamy
MAP: mitogen-activated protein
MATα: Mating type *α*
MFA1: Mating type A1
*MF*α: Mating α factor precursor
MTL: Mating type locus
Myc: Myc epitope tag
NA: not analyzed
NADH: Nicotinamide adenine dinucleotide
Ofr1: Opaque Formation Regulator 1
orf19.3475: Candida gene *orf19*.*3475*
p: mating projection
pACTS: plasmid pACTS
pACT1: plasmid pACT1
PAS: Per-Arnt-Sim
PI: propidium iodide
PST2: Protoplasts-SecreTed 1
qRT-PCR: quantitative reverse transcription PCR
RNA-Seq: RNA sequencing
ROS: reactive oxidative species
SCD: synthetic complete medium
SDS: sodium dodecyl sulfate
SIS1: SIt4 Suppressor
SOD5: SuperOxide Dismutase 5
SSA2: Stress-Seventy subfamily A
STE2: STErile 2
TAP: Tandem affinity purification
TCA: tricarboxylic acid
tetON: tetracycline-induced
tetON-HSF1/hsf1: *tetON*-promoter–controlled conditional expression strain of *HSF1*
WOR1: White-Opaque Regulator 1
WT: wild type
Yci1: Dipeptidyl aminopeptidase Yci1
YPD-K: 1% yeast extract, 2% peptone, 2% glucose, 0.25% K_2_HPO_4_
YP-K: 1% yeast extract, 2% peptone, 0.25% K_2_HPO_4_
