# Supplementary figures and images for: Environment-induced same-sex mating in the yeast Candida albicans through the Hsf1–Hsp90 pathway
Source: PLoS Biol. 2019 Mar 13;17(3):e2006966. doi: 10.1371/journal.pbio.2006966 (PMC6415874; doi:10.1371/journal.pbio.2006966)

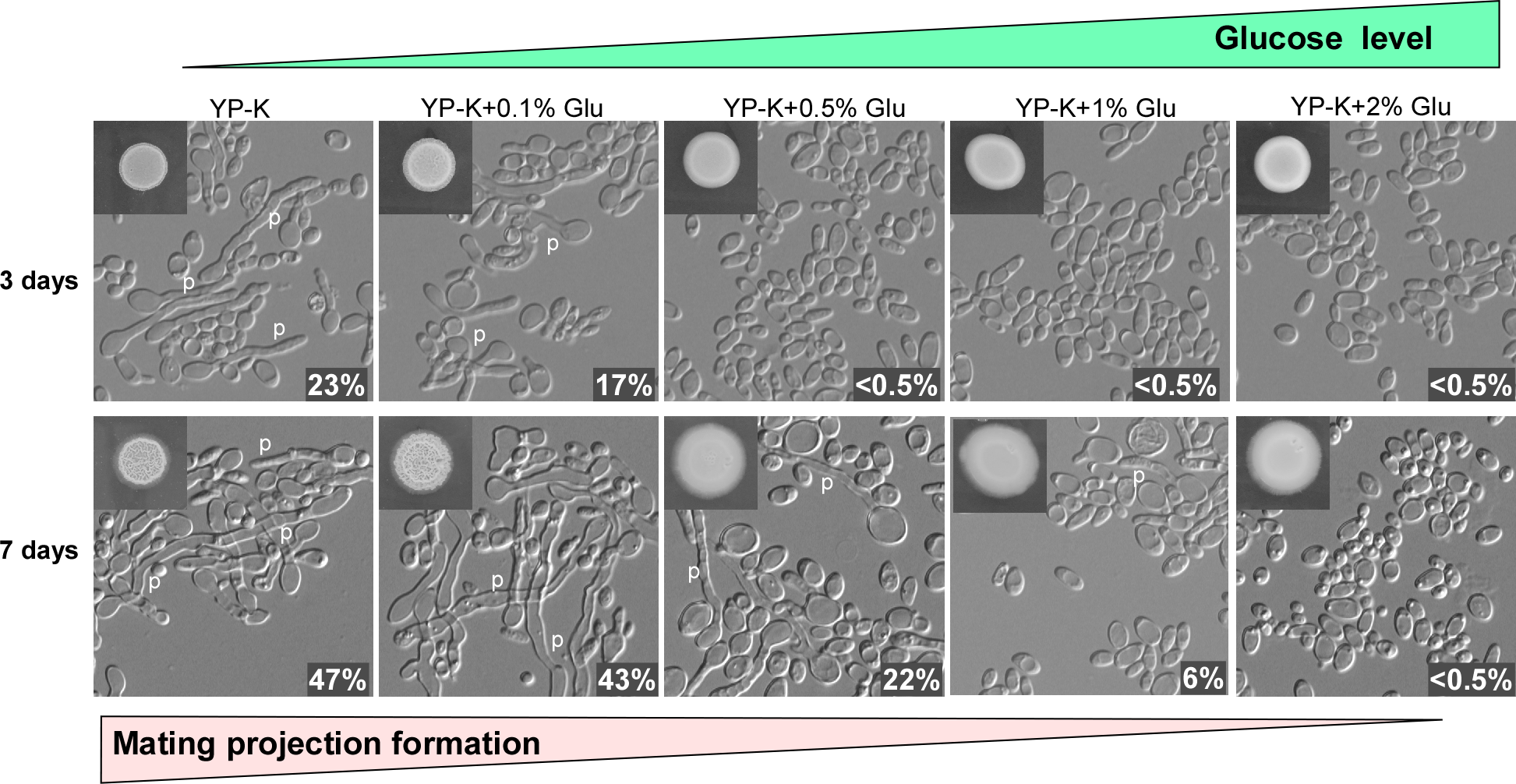

Supplement: S1 Fig — 1 × 10^7^ cells of strain GH1350a were spotted on media containing different levels of glucose and cultured at 25°C for three or seven days. Percentages of projected cells are indicated in the corresponding images. The percentage of projected cells decreases with the increase of glucose level. (TIF) [file pbio.2006966.s001.tif]

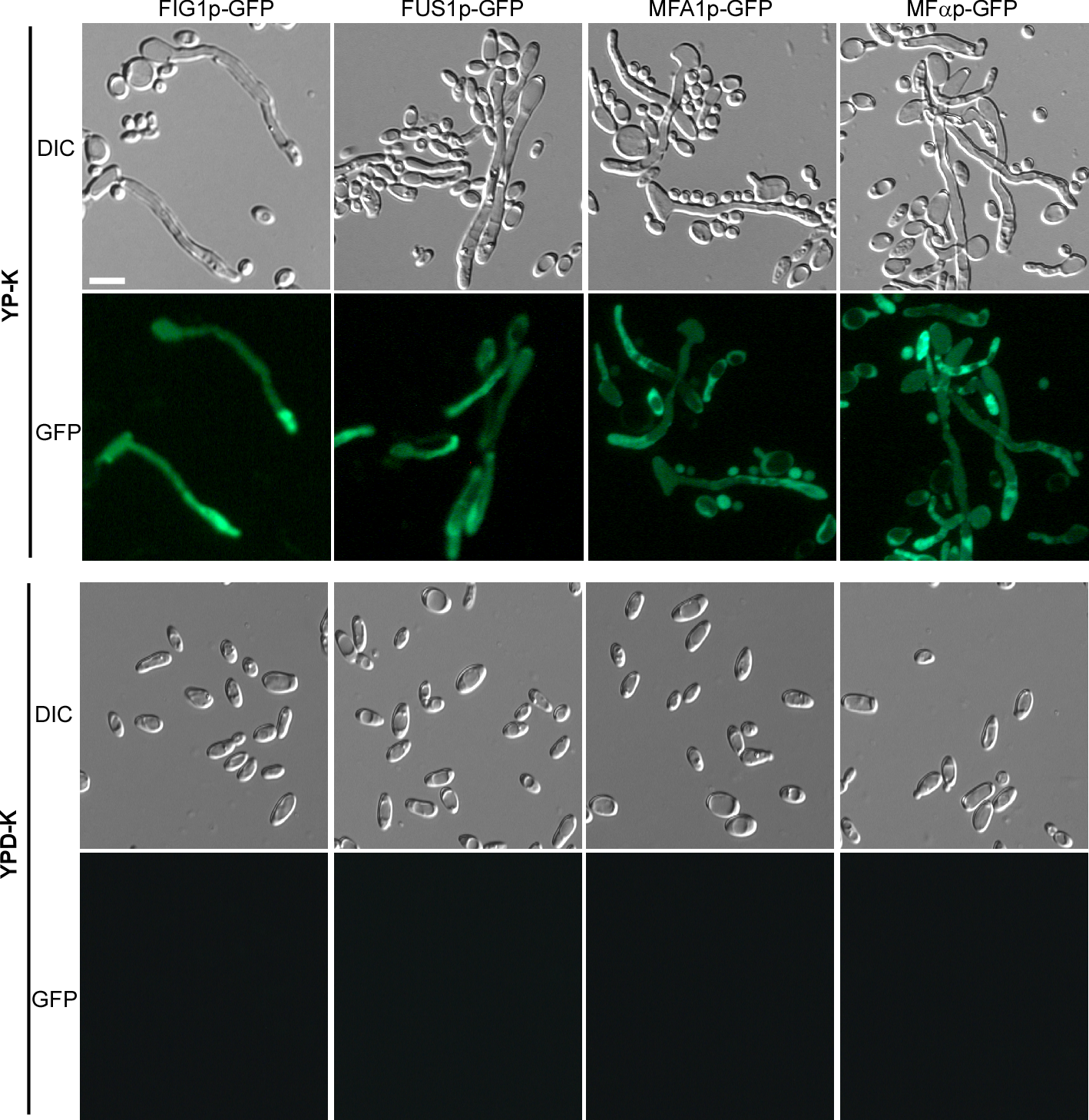

Supplement: S2 Fig — 1 × 10^5^ cells of each GFP-tagged strain (GH1013 background) were spotted on YP-K or YPD-K medium and cultured at 25°C for five days. Scale bar, 10 μm. DIC, differential interference contrast; GFP, green fluorescent protein; YPD-K, yeast extract-peptone-glucose-K2HPO4; YP-K, yeast extract-peptone-K2HPO4. (TIF) [file pbio.2006966.s002.tif]

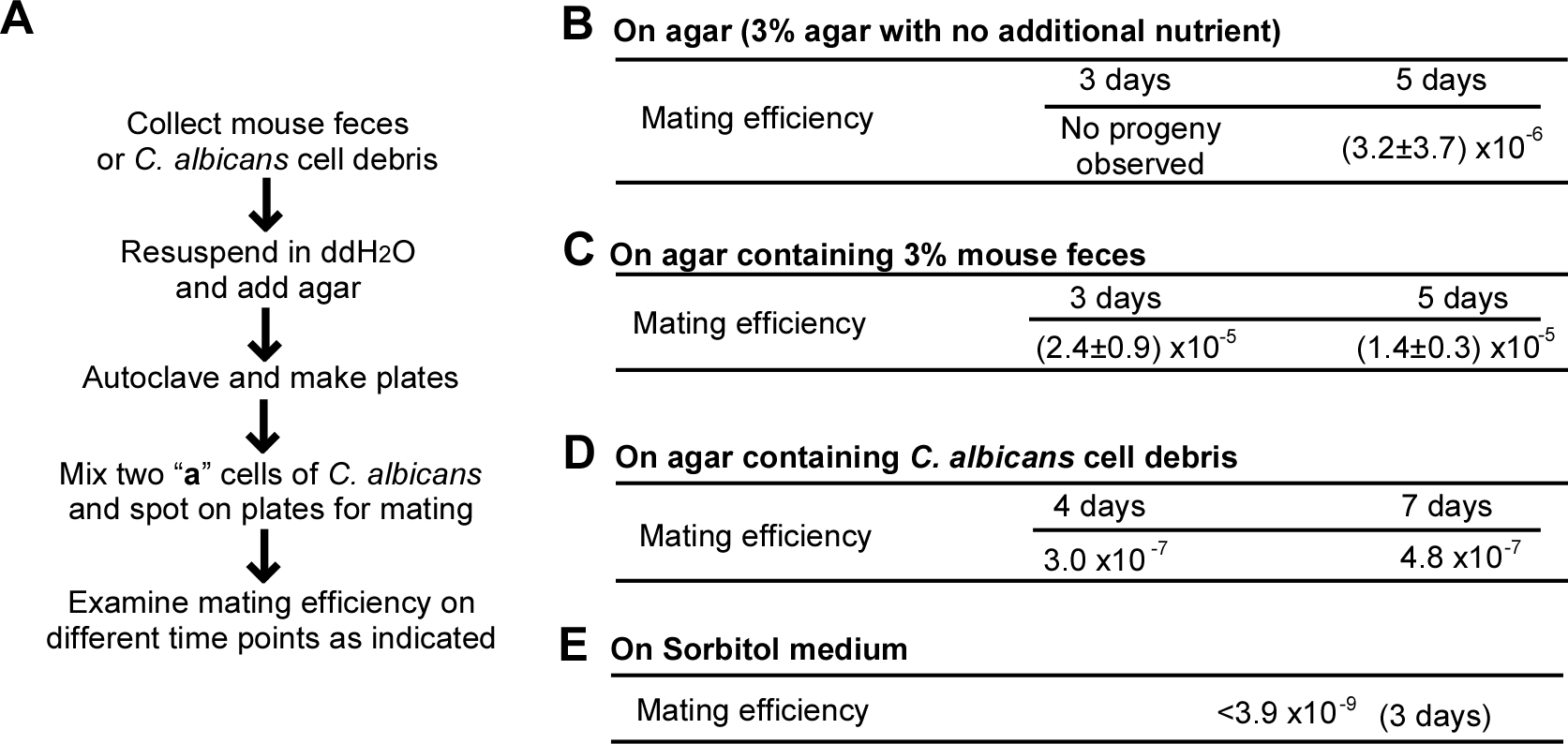

Supplement: S3 Fig — (A) Diagram of experimental procedures. (B, C, and D) Mating efficiency on 3% agar without additional nutrients (B), on agar containing 3% mouse feces (C), and agar containing C. albicans debris (D). 1 × 10^7^ cells of GH1013 and 1 × 10^7^ cells of GH1350a were mixed and cultured on different medium plates at 25°C for three to seven days. Mating mixtures were replated onto SCD-Arg, SCD-His, and both dropout plates for selectable growth and mating efficiency calculation. For mating on agar without additional nutrients (B), a portion of cells underwent cell death and released nutrients for the survived cells. (E) Mating on sorbitol medium (opaque filamentation inducing medium). The numerical data are presented in S3 Data. Arg, arginine; His, histidine; SCD, synthetic complete medium. (TIF) [file pbio.2006966.s003.tif]

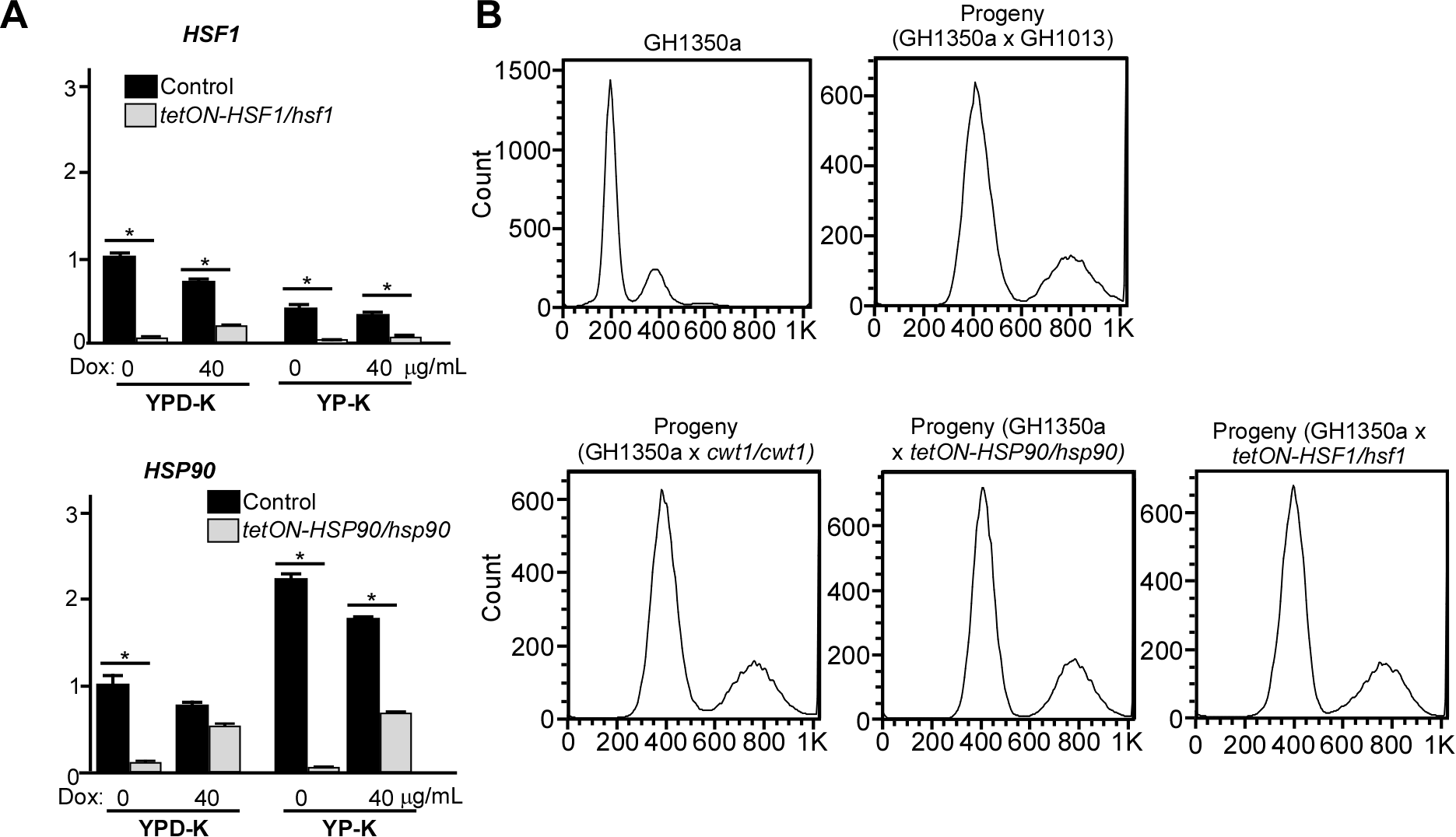

Supplement: S4 Fig — (A) Relative transcriptional expression levels of HSF1 or HSP90 in the control and tetON-HSF1/hsf1 or tetON-HSP90/hsp90 mutant on YP-K and YPD-K media with or without doxycycline (40 μg/mL). 1 × 10^5^ cells were spotted on YP-K or YPD-K medium and cultured at 25°C for three days. Error bars, standard errors of technical duplicates *p < 0.05, two-tailed Student t test. Experiment was performed in biological replicate and representative image is shown. (B) FACS analysis of the DNA content of progeny strains. Parental strain GH1350a used as a diploid control. Mating progeny contain DNA content corresponding to 4C and 8C peaks confirming their tetraploid nature. This figure is related to the quantitative results presented in supplementary S1 Table. The numerical data are presented in S3 Data. FACS, Fluorescence-activated cell sorting; Hsf1, Heat Shock transcription Factor 1; Hsp90, Heat shock protein 90; tetON, tetracycline-induced; tetON-HSF1/hsf1, tetON-promoter–controlled conditional expression strain of HSF1; YPD-K, yeast extract-peptone-glucose-K2HPO4; YP-K, yeast extract-peptone-K2HPO4. (TIF) [file pbio.2006966.s004.tif]

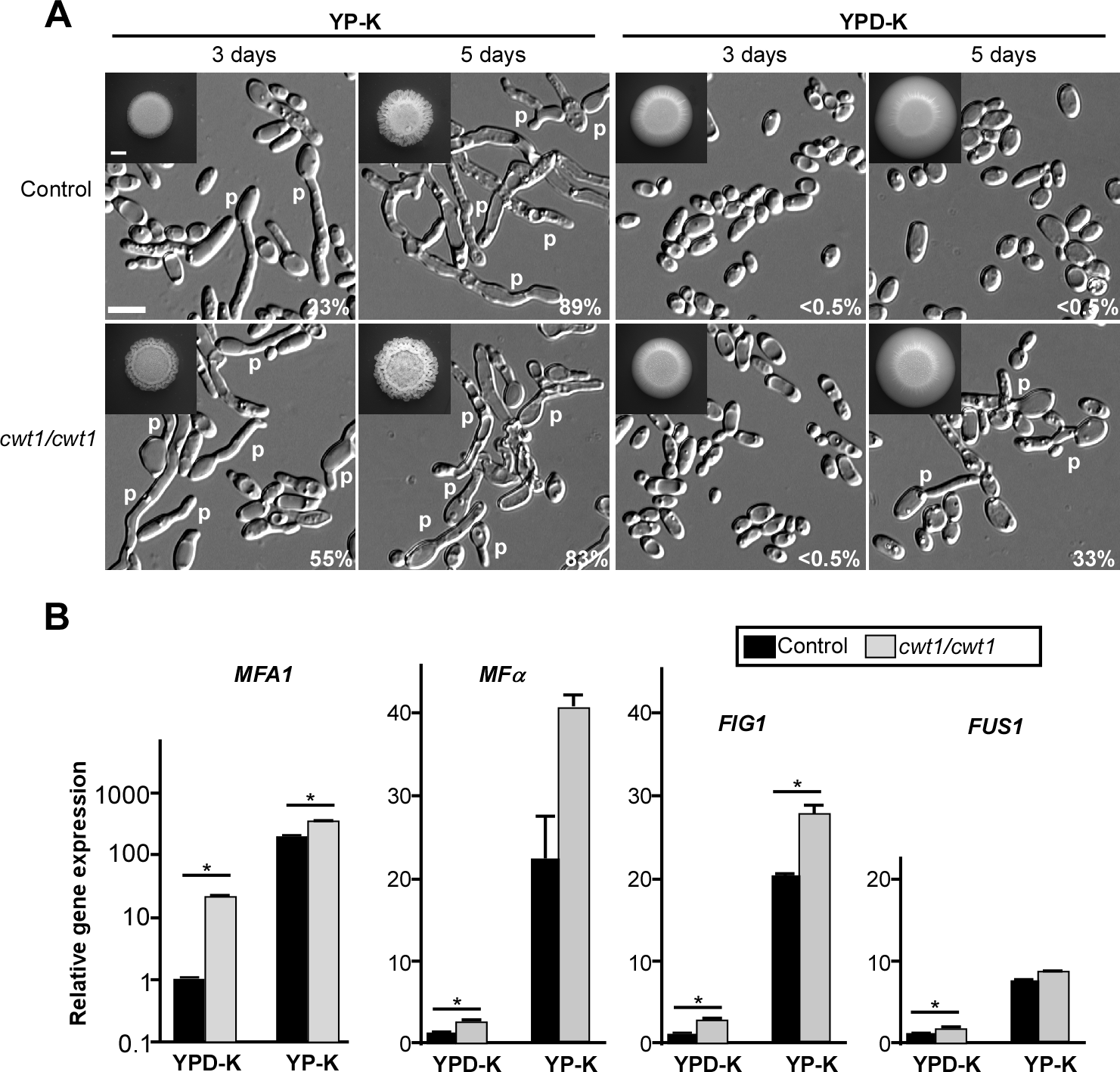

Supplement: S5 Fig — (A) Formation mating projections in the cwt1/cwt1 mutant on YPD-K and YP-K media. 1 × 10^5^ cells of each strain were spotted on different media and cultured at 25°C for three or five days. Scale bar for colonies, 2 mm; scale bar for cells, 10 μm. (B) Relative expression levels of mating-related genes in the control (GH1350a) and cwt1/cwt1 mutant on YPD-K and YP-K media. Error bars, standard errors. *p < 0.05, two-tailed Student t test. Two biological and two technical repeats were performed, respectively. The numerical data are presented in S3 Data. Cwt1, Cell Wall Transcription factor 1; p, mating projection; YPD-K, yeast extract-peptone-glucose-K2HPO4; YP-K, yeast extract-peptone-K2HPO4. (TIF) [file pbio.2006966.s005.tif]

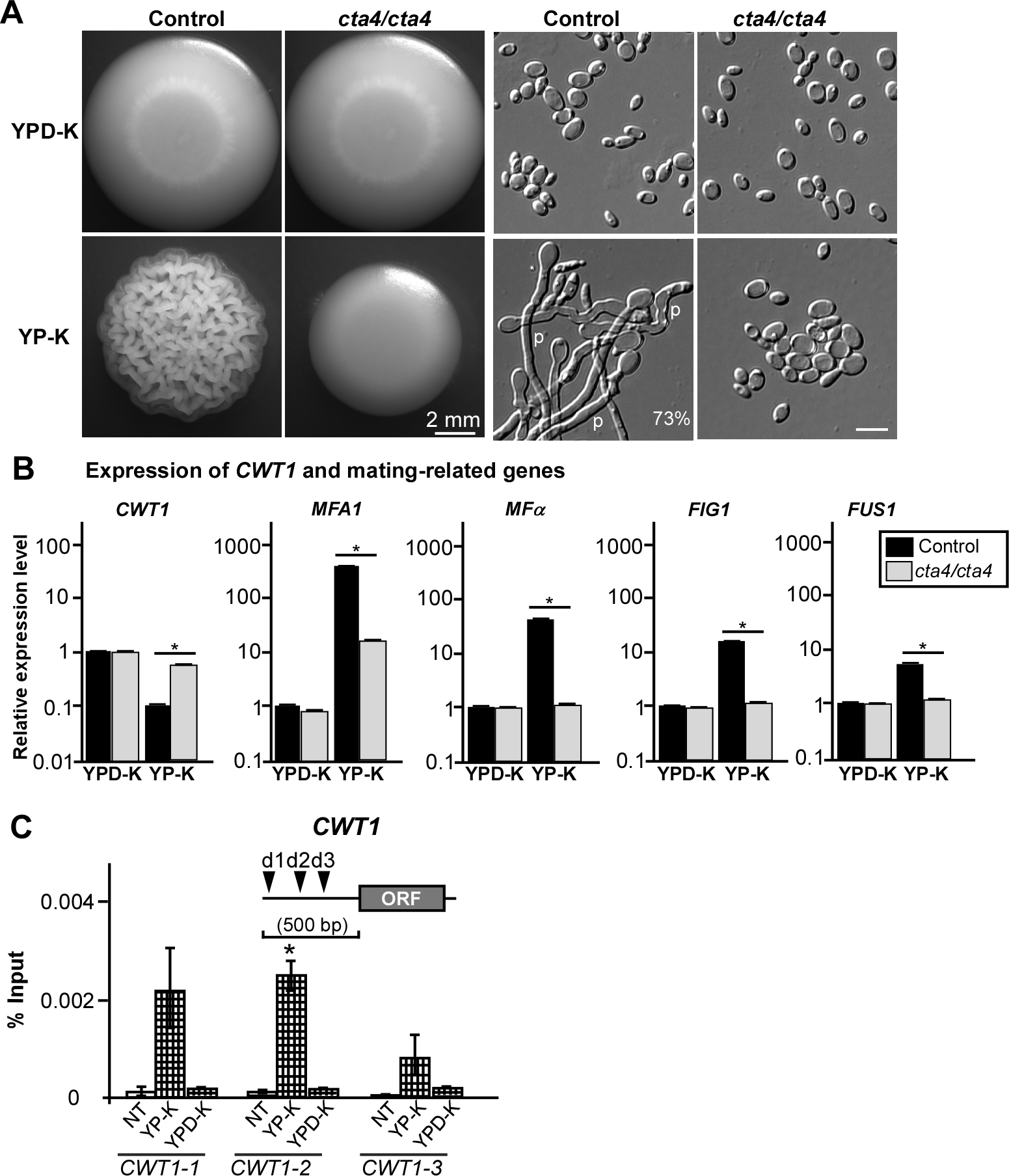

Supplement: S6 Fig — (A) Colony and cellular morphologies of the control (WT, GH1350a) and cta4/cta4 mutant on YP-K medium. 1 × 10^5^ cells of each strain were spotted on different media and cultured at 25°C for five days. Scale bar for colonies, 2 mm; scale bar for cells, 10 μm. (B) Relative expression levels of CWT1 and mating-related genes in the control (GH1350a) and cta4/cta4 mutant on YPD-K and YP-K media. Cells of C. albicans used for qRT-PCR assays were cultured at 25°C for five days. Error bars, standard errors. *p < 0.05, two-tailed Student t test. Two biological and two technical repeats were performed, respectively. (C) Cta4 binds to the promoters of CWT1. ChIP assays were performed in TAP-tagged Cta4 strains. Cells of C. albicans used for ChIP assays were grown on YP-K or YPD-K medium at 25°C for 24 hours. Percentages of input genomic DNA are indicated. Dark arrows indicate detected promoter regions. d1, d2, and d3, three detected sites of CWT1. Error bars represent standard error of two technical replicates. *p < 0.05, two-tailed Student t test. Experiment was performed in biological replicate with a representative image shown. The numerical data are presented in S3 Data. ChIP, chromatin immunoprecipitation; Cta4, Candida TransActivating protein 4; Cwt1, Cell Wall Transcription factor 1; p, mating projection; qRT-PCR, quantitative reverse transcription PCR; WT, wild type; YPD-K, yeast extract-peptone-glucose-K2HPO4; YP-K, yeast extract-peptone-K2HPO4. (TIF) [file pbio.2006966.s006.tif]
