# Supplementary material for: Environment-induced same-sex mating in the yeast Candida albicans through the Hsf1–Hsp90 pathway
Source: PLoS Biol. 2019 Mar 13;17(3):e2006966. doi: 10.1371/journal.pbio.2006966 (PMC6415874; doi:10.1371/journal.pbio.2006966)
Supplement: S1 Table — Cwt1, Cell Wall Transcription factor 1; Hsf1, Heat Shock transcription Factor 1; Hsp90, Heat shock protein 90; tetON, tetracycline-induced; tetON-HSF1/hsf1, tetON-promoter–controlled conditional expression strain of HSF1. (DOC) [file pbio.2006966.s007.doc]

**Table S1. Efficiency of same-sex mating in the *tetON-HSF1/hsf1* and *cwt1/cwt1* mutants.**

| **Cross** | | **YP-K** | | **YPD-K** | |
| --- | --- | --- | --- | --- | --- |
|  |  | Doxycycline (μg/mL) | | Doxycycline (μg/mL) | |
|  |  | 0 | 40 | 0 | 40 |
| WTa1 | WTa2 | (4.7±0.5)×10^-6^ | (9.1±0.3)×10^-7^ | <2.8×10^-9^ | <4.5×10^-9^ |
| WTa1 | *hsf1/tetON-HSF1* | (2.5±0.1)×10^-6^ | (1.8±0.2)×10^-6^ | (6.8±5.0)×10^-8^ | <3.5×10^-9^ |
| WTa1 | *cwt1/cwt1-1* | (5.2±0.6)×10^-6^ | NA | (1.2±1.0)×10^-7^ | NA |
| WTa1 | *hsp90/tetON-HSP90* | 2.5×10^-6^ | (3.4±1.0)×10^-5^ | <4.2×10^-7^ | <1.5×10^-7^ |
| *cwt1/cwt1-1* | *cwt1/cwt1-2* | (2.2±0.5)×10^-6^ | NA | (1.3±0.9)×10^-7^ | NA |

**Notes:** 1 x 10^7^ cells of each mating partner strain were mixed and cultured on YP-K or YPD-K medium with or without doxycyline at 25°C for five days (except for the WTa1 x *hsp90/tetON-HSP90* cross that was cultured for seven days). Mating mixtures were then replated onto selection media to determine the mating frequency. WTa1, GH1350a (*arg4-*); WTa2, GH1013 containing a cartTA cassett (*his1-*); *hsf1/tetON-HSF1, HSF1* conditional knockout mutant *(his1-); cwt1/cwt1-1,* a *CWT1* mutant *(ura3-)* generated from *GH1013*; *cwt1/cwt1-2,* a *CWT1* mutant *(arg4-)* generated from GH1350a; *tetON-HSP90/hsp90,* a *tetON* promoter-controlled conditional knockout strain of *HSP90* with an ectopically expressed *WOR1* (+*pACT1*-*WOR1*). To increase the expression level of *cartTA* in opaque cells, the cassette was integrated into the opaque-specific *OP4* locus in the *tetON-HSP90/hsp90* mutant by transforming with fusion PCR products of the *cartTA-Cahph*.

NA, not analyzed.
